# DB-2B, a Novel and Selective STAT3 Inhibitor Inhibits Colorectal Cancer Progression In Vitro and In Vivo

**DOI:** 10.3390/biom16050752

**Published:** 2026-05-20

**Authors:** Yuting Chen, Dianyang Li, Mengdi Zhang, Zhixia Qiu, Honghe Zhang, Wenying Yu, Zhiyong Liang, Maode Lai

**Affiliations:** 1 Department of Pathology, Peking Union Medical College Hospital, Chinese Academy of Medical Science & Peking Union Medical College, Beijing 100730, China; 2 Research Unit of Intelligence Classification of Tumor Pathology and Precision Therapy, Chinese Academy of Medical Sciences (2019RU042) & Zhejiang University School of Medicine, Hangzhou 310058, China; 3 Department of Pathology, Zhejiang University School of Medicine, Hangzhou 310058, China; 4 School of Basic Medicine and Clinical Pharmacy, China Pharmaceutical University, Nanjing 210009, China; 5 Department of Pharmacy, Nanjing Drum Tower Hospital, China Pharmaceutical University, Nanjing 210009, China; 6 Department of Pharmacology, School of Pharmacy, China Pharmaceutical University, Nanjing 210009, China; 7 State Key Laboratory of Natural Medicines, China Pharmaceutical University, Nanjing 210009, China

**Keywords:** STAT3 inhibitor, SH2 domain, small molecule inhibitor, colorectal cancer, DB-2B, anticancer, stemness, oral administration

## Abstract

Activation of signal transducer and activator of transcription 3 (STAT3) is implicated in tumor progression and correlates with poor prognosis and reduced survival. In colorectal cancer (CRC), STAT3 activation serves as a key indicator of unfavorable outcomes. However, the scarcity of clinically available STAT3 inhibitors hinders the development of personalized treatment strategies targeting STAT3. Therefore, we aimed to develop a novel STAT3 inhibitor based on the molecular structure of STAT3 and our previously reported STAT3 inhibitor LY17 to inhibit the progression of CRC. The binding of the novel STAT3 inhibitor DB-2B to STAT3 was confirmed by computational docking, surface plasmon resonance, isothermal titration calorimetry, and cellular thermal shift assays. Western blotting and immunofluorescent staining demonstrated that DB-2B specifically inhibited STAT3 activation and nuclear translocation. In vitro studies revealed that DB-2B significantly suppressed proliferation, induced apoptosis, arrested cell cycle progression, and attenuated stemness by inhibiting STAT3 activation and its downstream signaling pathways. In vivo, DB-2B exhibited favorable oral bioavailability and safety, while significantly inhibiting the progression of CRC. Collectively, this study presents DB-2B as a promising small-molecule STAT3 inhibitor for the targeted treatment of CRC.

## 1. Introduction

Signal transducer and activator of transcription 3 (STAT3), a key member of the STAT family, serves as a critical mediator of cytokine-induced gene transcription [1,2]. Structurally, STAT3 is a modular protein composed of several functional domains: the amino-terminal, coiled-coil, DNA-binding, linker, Src-homology 2 (SH2), and trans-activation domains [3]. Among these, the SH2 domain is functionally pivotal. It facilitates the specific recognition of phosphorylated tyrosine residues on cytokine receptors, which function as docking sites for cytoplasmic STAT3 following activation by Janus kinases (JAKs) [2,4]. This recruitment brings STAT3 into proximity with JAKs, facilitating the phosphorylation of STAT3 at Tyr705. Crucially, this phosphorylation triggers a reciprocal interaction where the SH2 domain of one monomer binds to the phosphorylated Tyr705 residue of another, driving STAT3 homo- or heterodimerization. Following dimerization, the complex translocates to the nucleus to regulate the expression of target genes involved in proliferation and apoptosis. Abnormal STAT3 expression has been shown to drive uncontrolled growth and progression in various tumors, including colorectal cancer (CRC) [2,5,6]. Moreover, STAT3 has been found to contribute to the maintenance of a cancer stem cell (CSC)-like phenotype in various cancers, including CRC, which is closely associated with chemoresistance and tumor relapse [7,8,9]. Collectively, the pivotal biological functions of STAT3 in cancer represent a major challenge for cancer therapy and pose a significant threat to patient survival.

CRC is one of the most commonly diagnosed cancers, accounting for approximately 8% of all newly diagnosed cancer cases [10]. The prognosis of CRC patients, especially those suffering from metastatic CRC, is generally poor, with a five-year survival rate of less than 20% [11]. For patients with metastatic CRC who experience disease progression following first-line chemotherapy or targeted therapy, effective therapeutic options are limited. Achieving a survival benefit exceeding three months remains a significant challenge in the management of refractory metastatic CRC. Consequently, personalized treatment strategies guided by tumor molecular characteristics may offer improved therapeutic prospects. Given the critical role of STAT3 in CRC progression and the fact that elevated STAT3 expression and phosphorylation are both associated with poorer prognosis, targeting STAT3 offers a promising therapeutic strategy to improve the clinical outcomes of CRC patients [12,13,14].

One approach to inhibit STAT3 is to target the upstream effectors involved in STAT3 activation, such as JAKs (e.g., Ruxolitinib) or IL-6 (e.g., Siltuximab) [15,16,17,18]. However, a direct inhibitor of STAT3 represents a more specific intervention strategy. Although several STAT3 inhibitors have shown promising anticancer effects in vitro and some have entered clinical trials, challenges such as poor solubility, low permeability, limited selectivity, and severe adverse effects have hindered their clinical advancement [19]. For instance, the STAT3 inhibitor Napabucasin (BBI-608) received orphan drug designation for gastric and pancreatic cancers but was later withdrawn. Despite demonstrating an acceptable safety profile in early-phase trials, Napabucasin failed to significantly improve overall survival in Phase III trials [20,21,22,23,24,25,26]. Thus, the limitations of the currently available STAT3 inhibitors underscore the urgent need to develop novel STAT3 inhibitors with improved efficacy, safety, and specificity for CRC therapy.

To address this need, we aimed to develop a novel STAT3 inhibitor as a potential therapeutic option for CRC. LY17, a STAT3 inhibitor we developed previously, showed promising anticancer effects in triple-negative breast cancer [27]. However, the 1,4-dihydroquinone motif within the structure of LY17 functioned as a Michael acceptor, rendering the compound prone to non-specific reactivity toward cysteine residues in proteins [28,29]. To avoid promiscuous covalent binding, structural modification of LY17 is essential. Therefore, in this study, we designed a STAT3 inhibitor DB-2B based on the molecular structure of the STAT3 SH2 domain and our previously developed STAT3 inhibitor LY17. DB-2B exhibited specificity in targeting STAT3 activation, which acted to inhibit cell growth, proliferation, migration, invasion, and stemness of CRC, while promoting apoptosis and cell cycle arrest. Oral gavage of DB-2B, which exhibited favorable pharmacokinetic properties and bioavailability, significantly suppressed CRC progression in vivo, demonstrating its potential as a promising therapeutic agent for CRC.

## 2. Materials and Methods

### 2.1. Cell Culture and Reagents

CRC cells, including RKO, DLD1, HT29, HCT8, HCT116, SW480 and SW620, were all purchased from American Type Culture Collection (ATCC, Manassas, VA, USA). DLD1, HT29, HCT8, HCT116, SW480 and SW620 cells were cultured in RPMI-1640 (Gibco, Carlsbad, CA, USA) supplemented with 10% fetal bovine serum (FBS, Gibco, Carlsbad, CA, USA) and 1% penicillin-streptomycin (Gibco, Carlsbad, CA, USA). RKO was cultured in DMEM (Gibco, Carlsbad, CA, USA) containing 10% FBS and 1% penicillin-streptomycin. A humidified incubator set at 37 °C and 5% CO_2_ was used to culture all cell lines.

TTI-101 was purchased from MCE (Shanghai, China). All compounds used for in vitro studies were dissolved in DMSO (MCE, Shanghai, China). To ensure rigorous experimental control, DMSO was added at a final concentration of 0.1% to the vehiclecontrol group. All antibodies used in this study and their respective concentrations are listed in Table S1.

### 2.2. Computational Docking of DB-2B

Computational docking was performed using Autodock tools (version 1.5.7) with default settings. The binding models of unphosphorylated STAT3 binding to double-stranded DNA (PDB code: 4E68) and DB-2B were constructed. The docking box was set to encompass the whole SH2 domain. Docking simulations were carried out using Autodock vina (version 1.1.2), with the exhaustiveness parameter set to 8. The resulting docking poses were visualized with Pymol (version 2.6), and the 2-D interaction diagrams were generated using Maestro [30,31].

### 2.3. Expression and Purification of Recombinant STAT3

Expression and purification of recombinant STAT3 protein were performed as previously described [32]. Briefly, the sequence of STAT3 (residues 132–716) was subcloned into the pET-28a vector and transformed into *Escherichia coli*. Protein expression was induced by overnight incubation with 0.5 mM Isopropyl β-D1-thiogalactopyranoside (IPTG) at 18 °C. Cell lysates were centrifuged to isolate the soluble fraction, which was subsequently purified using a Ni-NTA column (Smart-Lifesciences, Changzhou, China) and a Cytiva Superdex™ 75 Increase column. The elution fraction containing STAT3 was concentrated to 10 mg/mL and dialyzed into a buffer containing 20 mM Hepes (pH = 9.0), 500 mM NaCl and 10% glycerol.

### 2.4. Surface Plasmon Resonance

Recombinant STAT3 protein was diluted to 100 μg/mL in sodium acetate buffer (10 mM, pH = 5.0) and immobilized on the Sensor Chip CM5 (Cytiva, Uppsala, Sweden). The binding affinity of DB-2B to STAT3 was analyzed using the Biacore S200 system (Cytiva, Uppsala, Sweden). A low-molecular-weight kinetic method was used, with a flow rate of 30 μL/min, 30 s for the binding phase, and 120 s for the dissociation phase. The K_D_ value was calculated with the Biacore S200 evaluation software.

### 2.5. Isothermal Titration Calorimetry (ITC)

ITC was conducted in buffer containing 10 mM Hepes (pH = 7.5), 50 mM NaCl, 1 mM EDTA and 3% DMSO utilizing a MicroCal PEAQ-ITC instrument (Malvern Panalytical, Malvern, UK), as previously described [32]. DB-2B and recombinant STAT3 protein were diluted in the same buffer at concentrations of 300 and 30 μM, respectively. The recombinant STAT3 protein was loaded into the sample cell and titrated with DB-2B. Following titration, the thermal parameter of DB-2B binding to STAT3 was analyzed using the MicroCal PEAQ-ITC analysis software (version 1.41, Malvern Panalytical, Malvern, UK).

### 2.6. Cellular Thermal Shift Assay (CETSA)

HCT116 cells were incubated in 8 mL of complete medium containing DB-2B or DMSO for 4 h. Following collection and resuspension in PBS containing phenylmethanesulfonyl fluoride (PMSF, Servicebio, Wuhan, China), the cell suspension was divided into 12 aliquots. The aliquots were then heated at a gradient of temperatures ranging from 37 to 52 °C for 3 min. Subsequently, cells were subjected to lysis through three rounds of alternating exposure to liquid nitrogen and a 37 °C heating block. The supernatant was separated by centrifugation at 20,000× *g* for 20 min, resuspended in loading buffer, and subsequently boiled for protein analysis.

### 2.7. Cell Proliferation Assay

A cell proliferation assay was performed using the Cell Counting Kit-8 (CCK-8, Biosharp, Beijing, China). Six hours after seeding in 96-well plates, 10 μL of CCK-8 reagent was added to each well, followed by a further 2 h incubation. Absorbance at 450 nm was measured using a Thermo Scientific™ Varioskan™ Flash Multimode Reader (Thermo Fisher Scientific, Waltham, MA, USA) and recorded as baseline (0 h or Day 0) absorbance. Subsequently, various concentrations of DB-2B were added, and absorbance was measured at the indicated time points using the same method.

### 2.8. Colony Formation Assay

DLD1 and HCT116 cells were inoculated into 6-well plates (2000 cells/well) and allowed to adhere for 6 h prior to exposure to DB-2B. Following a growth period of 7–14 days, colonies formed were fixed with 4% paraformaldehyde (PFA; Biosharp, Beijing, China) for 1 h, rinsed, and subsequently stained using crystal violet. Colonies consisting of more than fifty cells were counted.

### 2.9. 5-Ethynyl-2’-deoxyuridine (EdU) Proliferation Assay

DLD1 and HCT116 cells were inoculated into 96-well plates (10,000 cells/well). After attachment, cells were exposed to gradient concentrations of DB-2B for a 24 h period. The EdU proliferation assay was evaluated utilizing the BeyoClick™ EdU-555 Cell Proliferation Assay Kit (Beyotime, Shanghai, China) in strict accordance with the recommended procedures. The proportion of EdU-positive cells was visualized and quantified using an Olympus LX53 inverted fluorescence microscope (Olympus, Tokyo, Japan).

### 2.10. Transwell Migration and Invasion Assay

For the transwell migration assays, 100,000 cells suspended in serum-free medium were seeded into the chambers of Transwell^®^ inserts containing 8 μm pores (Corning, New York, NY, USA). Medium containing 10% FBS was placed in the bottom wells. Non-migrated cells were swabbed away, and the migrated cells on the bottom of the insert membrane were fixed and stained with crystal violet. Each transwell insert was rinsed and air-dried before counting the migrated cells under a light microscope.

For the transwell invasion assay, the Transwell^®^ insert membrane was coated with Matrigel (Corning, New York, NY, USA), diluted at a ratio of 1:20. The Transwell^®^ insert was solidified at 37 °C for 30 min before cell seeding. The following steps were identical to those described for the transwell migration assay.

### 2.11. Spheroid Formation Assay

To assess spheroid formation ability, cells were first filtered through a 40 μm cell strainer and then sorted into ultra-low attachment 96-well plates (Corning, New York, NY, USA). FACS Aria III (BD Biosciences, San Jose, CA, USA) was used to sort and seed a single cell into each well pre-loaded with 100 μL of complete spheroid culture medium consisting of DMEM/F12 (Gibco, Carlsbad, CA, USA) supplemented with 1× B27 (Invitrogen, Carlsbad, CA, USA), 20 ng/mL EGF (PeproTech, Rocky Hill, NJ, USA), and 10 ng/mL bFGF (Sino Biological, Beijing, China). Cells were cultured for 7–14 days until the spheroids reached a diameter of approximately 100 μm. Then, the medium was replaced with the spheroid culture medium described above, supplemented with either DB-2B or DMSO. Ten spheroids of similar diameter were selected per group. The diameter of the spheroids was recorded for the subsequent 5 days following DB-2B addition. The spheroid-forming efficiency (SFE) was calculated as the percentage of spheroids with a diameter greater than 200 μm.

### 2.12. Flow Cytometry

For cell cycle analysis, cells were first synchronized by serum starvation in serum-free medium for 24 h. After DB-2B treatment, cells were harvested and fixed by dropwise addition into pre-cooled (−20 °C) anhydrous ethanol under vigorous stirring, and incubated overnight at −20 °C. Samples were stained using the Cell Cycle Staining Kit (Multi Sciences, Hangzhou, China) and analyzed for cell cycle distribution using the CytoFLEX flow cytometer (Beckman Coulter, Brea, CA, USA).

For cell apoptosis detection, an Annexin V-FITC/PI Apoptosis Detection Kit (Vazyme, Nanjing, China) was used according to the manufacturer’s instructions. Briefly, cells treated with DB-2B for 48 h were harvested and stained with the annexin V-FITC and PI staining solution for 10 min. Cell apoptosis was analyzed using the CytoFLEX flow cytometer.

### 2.13. Immunofluorescence

Cells for immunofluorescence were seeded into the glass-bottom dishes (Biosharp, Beijing, China). Cells were fixed with 4% PFA for 10 min. A 15 min incubation with 0.1% Triton X-100 was carried out for permeabilization. To minimize non-specific binding, cells were blocked with 5% newborn bovine serum (NBS, Tianhang, Hangzhou, China). Cells were then incubated with primary antibodies, followed by appropriate secondary antibodies. Nuclei were counterstained with DAPI (Beyotime, Shanghai, China). Fluorescence images were acquired with an Olympus FV3000 confocal laser scanning microscope (Olympus Corporation, Tokyo, Japan).

### 2.14. Animal Experiments

For in vivo administration, DB-2B and TTI-101 were prepared in a vehicle comprising 2% DMSO, 40% PEG300, 5% Tween-80, and 53% saline. Control mice received the corresponding vehicle alone to ensure rigorous experimental control.

Pharmacokinetic studies were conducted using female SD rats (GemPharmatech, Nanjing, China) weighing approximately 180–200 g. Blood plasma samples were collected at specific time points following single intravenous (5 mg/kg, n = 3) and intragastric administration (50 mg/kg, n = 4). Plasma samples were frozen at −80 °C immediately after collection until High-Performance Liquid Chromatography-Tandem Mass Spectrometry (HPLC−MS/MS) analysis. Pharmacokinetic parameters were calculated using WinNonlin software (version 8.1).

To evaluate the in vivo toxicity of DB-2B, six-week-old female ICR mice were purchased from GemPharmatech (Nanjing, China). Mice were randomly divided into 2 groups and administered either vehicle or DB-2B (100 mg/kg) via oral gavage once daily for 5 consecutive days. Body weight, average food intake, and average water consumption were recorded daily. At the experimental endpoint, the heart, lungs, liver, spleen and kidneys of each mouse were dissected after the mice were euthanized.

For the subcutaneous xenograft model, six-week-old female BALB/c nude mice were purchased from GemPharmatech (Nanjing, China). A total of 2 × 10^6^ DLD1 cells were inoculated subcutaneously into the mouse flanks. Upon reaching a tumor volume of approximately 80 mm^3^, mice were randomly divided into 5 groups. Each group received oral administration of vehicle, 20 mg/kg TTI-101, 10 mg/kg DB-2B, 20 mg/kg DB-2B, or 50 mg/kg DB-2B, respectively, once daily for 14 days. Tumor volume and body weight were measured every other day by investigators who were blinded to the specific treatment allocations to prevent observational bias. Tumor volume was calculated using the following formula: tumor volume (mm^3^) = (length × width^2^)/2. Tumors were dissected after the mice were euthanized and fixed in 4% PFA after weighing.

Animals were kept under specific pathogen-free (SPF) conditions at the Laboratory Animal Center of Zhejiang University. All experimental procedures involving animals were approved by the Ethics Committee for Animal Welfare of Zhejiang University (ZJU20240260). Sample size was chosen based on the ‘Reduction’ principle of the 3Rs (Replacement, Reduction, Refinement) to ensure statistical significance using the minimum number of animals.

### 2.15. Statistical Analysis

All statistical analyses were performed using GraphPad Prism software (version 10.0, GraphPad Software, San Diego, CA, USA). Data with error bars represent means ± standard deviation (SD). All experiments were performed with at least 3 independent replicates. Comparisons between groups were analyzed using the unpaired Student’s *t*-test or one-way analysis of variance (ANOVA). Statistical significance was denoted by *p*-values (* *p* < 0.05, ** *p* < 0.01, *** *p* < 0.001).

Detailed descriptions of additional materials and methods are provided in the Supplementary Materials.

## 3. Results

### 3.1. Design and Synthesis of the Novel STAT3 Inhibitor DB-2B

**Figure 1:**
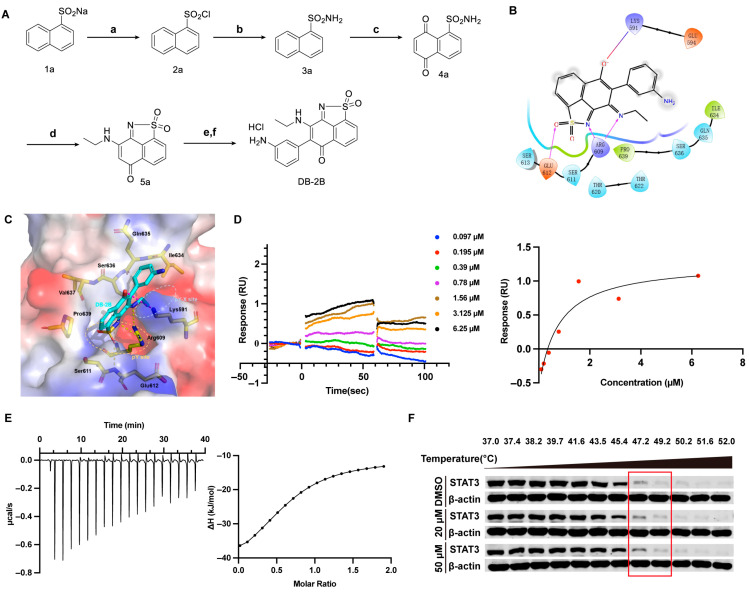
Design and Synthesis of the SH2 domain-targeting STAT3 inhibitor DB-2B. (**A**) Synthetic route of DB-2B. (**B**) 2D interaction diagram of DB-2B docked into the STAT3 SH2 domain. (**C**) Predicted 3D binding pose of DB-2B within the SH2 domain. (**D**) Surface plasmon resonance assay showing binding of DB-2B to purified STAT3, with a baseline equilibration phase prior to analyte injection (t < 0). (**E**) Isothermal titration calorimetry measurement of DB-2B binding to STAT3. (**F**) Cellular thermal shift assay showing DB-2B-induced stabilization of STAT3 in HCT116 cells. The red box highlights the temperature range in which an obvious change in the STAT3 signal can be observed. Original western blots can be found in the Supplementary Materials.

To optimize the potentially reactive chemical structure of LY17, we introduced methylamine substituents onto the 1,4-benzoquinone moiety to block Michael addition reactivity of LY17. Concurrently, the amine side chain was replaced with a phenyl group via C-C coupling, leading to the discovery of DB-2B (Figure 1A and Figures S1–S4). The structurally modified compound DB-2B was designed to target the SH2 domain of STAT3. The binding mode of DB-2B with STAT3 was predicted via molecular docking using Autodock Vina [33,34] (Figure 1B,C). The SH2 domain, which binds phosphorylated Tyr705 and facilitates STAT3 dimerization, was selected as the docking site. Docking results indicated that DB-2B interacted with the pY and pY-X pockets of the SH2 domain based on the crystal structure of unphosphorylated STAT3 bound to dsDNA (PDB code: 4E68) [35]. Specifically, insertion of the sulfonyl group into the pY site enabled hydrogen bond formation with the main chain of Glu612 and the side chain of Arg609, respectively. Meanwhile, another hydrogen bond between Arg609 and ethyl-substituted arylamine further stabilized the binding conformation of the tricyclic scaffold. Additionally, the quinone moiety formed a salt bridge with the side chain of Lys591, with the ethyl substituent fitting into the hydrophobic subpocket pY-X. To verify whether DB-2B, as predicted by computational docking, could specifically bind to STAT3 protein, we conducted additional experiments to explicitly determine the binding between DB-2B and STAT3. SPR assay confirmed that DB-2B bound to STAT3 with potent affinity, yielding a K_D_ value of 1.06 μΜ (Figure 1D). The interaction between STAT3 and DB-2B was further verified by ITC assay with a K_D_ value of 7.5 μΜ (Figure 1E). In addition, CETSA also confirmed the direct binding of DB-2B to STAT3, as DB-2B stabilized STAΤ3 against thermal denaturation (Figure 1F). Taken together, the novel STAT3 inhibitor DB-2B was demonstrated to be a direct STAT3 inhibitor.

### 3.2. DB-2B Inhibited STAT3 Activation in CRC Cells

**Figure 2:**
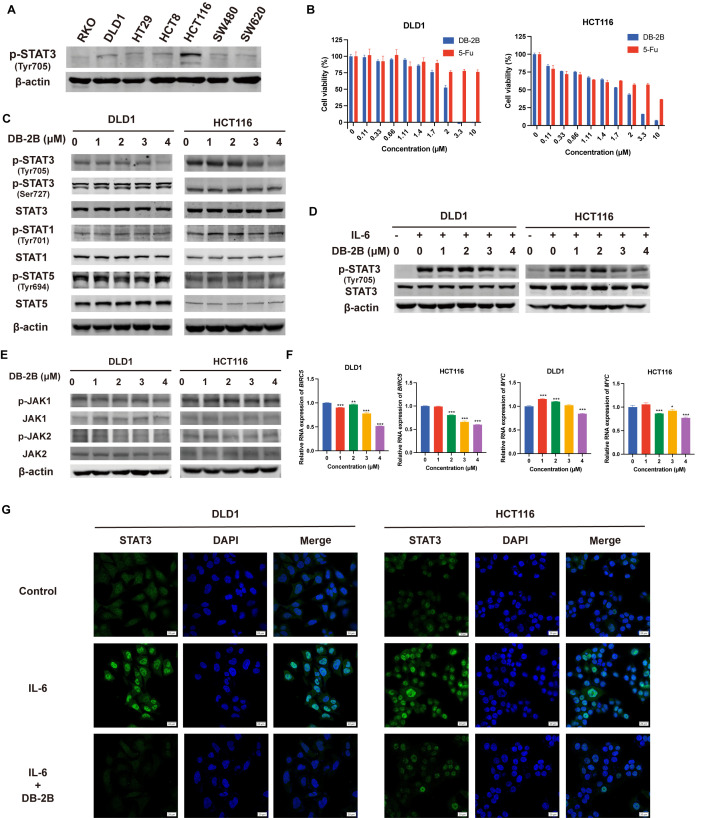
DB-2B inhibited STAT3 activation in colorectal cancer (CRC). (**A**) Phosphorylated STAT3 levels in CRC cell lines. (**B**) Cell viability of DLD1 and HCT116 cells following 24 h treatment with increasing concentrations of DB-2B and 5-fluorouracil (5-Fu). (**C**) The phosphorylation levels of STAT3, STAT1, and STAT5 following DB-2B treatment. (**D**) The phosphorylation levels of STAT3 in IL-6-stimulated DLD1 and HCT116 cells treated with DB-2B. (**E**) The phosphorylation of JAK1 and JAK2 in DB-2B-treated HCT116 and DLD1 cells. (**F**) Relative mRNA levels of *BIRC5* and *MYC* in HCT116 and DLD1 cells after DB-2B exposure. (**G**) The localization of STAT3 after DB-2B treatment in IL-6-stimulated HCT116 and DLD1 cells. Scale bar = 20 μm. *p*-values were calculated using one-way ANOVA. * *p* < 0.05, ** *p* < 0.01, *** *p* < 0.001.

To select appropriate models for evaluating the inhibitory effect of DB-2B, we first screened basal STAT3 phosphorylation levels in various CRC cell lines (Figure 2A). DLD1 and HCT116 cells exhibited higher basal STAT3 phosphorylation levels and were therefore selected for further studies. DB-2B demonstrated potent inhibitory activity in CRC cells, yielding 24 h IC_50_ values of 2.027 μM for DLD1 and 2.069 μM for HCT116, exhibiting significantly higher anti-tumor potency compared to the clinical chemotherapy drug 5-fluorouracil (5-FU) and our previously designed STAT3 inhibitor LY17 (Figure 2B and Figure S5). The phosphorylation status of STAT3 at an effective concentration of DB-2B was further examined. It was shown that the phosphorylation level of STAT3 at Tyr705 was effectively inhibited, while Ser727 phosphorylation was unaffected (Figure 2C). Meanwhile, DB-2B did not inhibit phosphorylation of other STAT family members, including STAT1 and STAT5 (Figure 2C). DB-2B also suppressed IL-6-stimulated STAT3 activation (Figure 2D). While STAT3 activation was inhibited, upstream JAK1 and JAK2 phosphorylation remained unchanged (Figure 2E). This suggested that DB-2B-induced inhibition of STAT3 activation was not due to altered activation states of the upstream JAK family. Furthermore, the mRNA expression of STAT3 target genes, including *BIRC5* and *MYC*, was downregulated following DB-2B treatment (Figure 2F). Immunofluorescence assays confirmed that DB-2B inhibited STAT3 nuclear translocation (Figure 2G), explaining the altered expression of STAT3-regulated genes. Collectively, DB-2B specifically inhibited the activation of STAT3 and its nuclear translocation, thereby blocking downstream gene transcription.

### 3.3. DB-2B Inhibited the Growth, Proliferation, Migration and Invasion of CRC Cells

**Figure 3:**
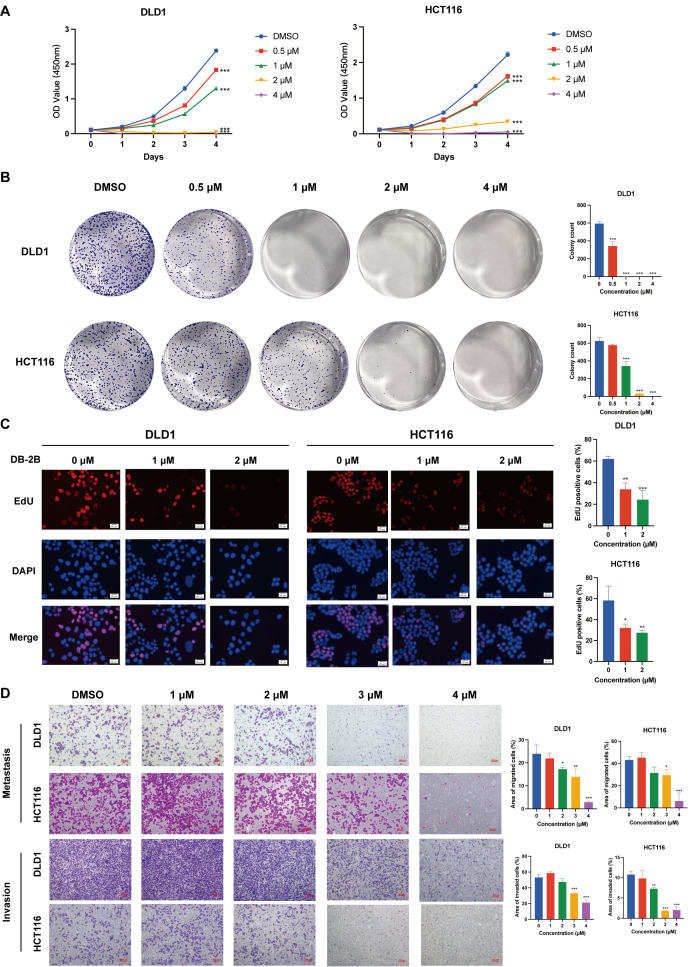
DB-2B inhibited colorectal cancer cell growth, proliferation, migration, and invasion in vitro. (**A**) Growth rate of DLD1 and HCT116 cells following DB-2B treatment measured by CCK-8 assay. (**B**) Formation of colonies of DLD1 and HCT116 cells under DB-2B treatment. (**C**) Proliferating cells labeled by EdU assay following DB-2B treatment. Scale bar = 20 μm. (**D**) Migration and invasion of HCT116 and DLD1 cells following DB-2B treatment assessed by Transwell migration and invasion assays. Scale bar = 100 μm. *p*-values were calculated using one-way ANOVA. * *p* < 0.05, ** *p* < 0.01, *** *p* < 0.001.

To evaluate the therapeutic potential of the novel STAT3 inhibitor DB-2B in CRC, we further investigated its effects on multiple biological functions in CRC cells. The growth rate of DLD1 and HCT116 cells was significantly inhibited by DB-2B (Figure 3A). DB-2B at concentrations of 0.5 μM and 1 μM significantly inhibited colony formation, whereas at higher concentrations, individual cells were unable to form colonies (Figure 3B). The EdU assay also revealed a significant decrease in proliferating cells following DB-2B treatment (Figure 3C). Additionally, Transwell assays were conducted to evaluate the migratory and invasive capacities of HCT116 and DLD1 cells following DB-2B treatment. The results showed that the migration and invasion capacities of CRC cells were significantly decreased following treatment with high concentrations of DB-2B (Figure 3D). Collectively, these results demonstrate that DB-2B suppresses CRC cell growth, proliferation, migration, and invasion in vitro.

### 3.4. DB-2B Induced Apoptosis and Suppressed Cell Cycle Progression in CRC Cells

**Figure 4:**
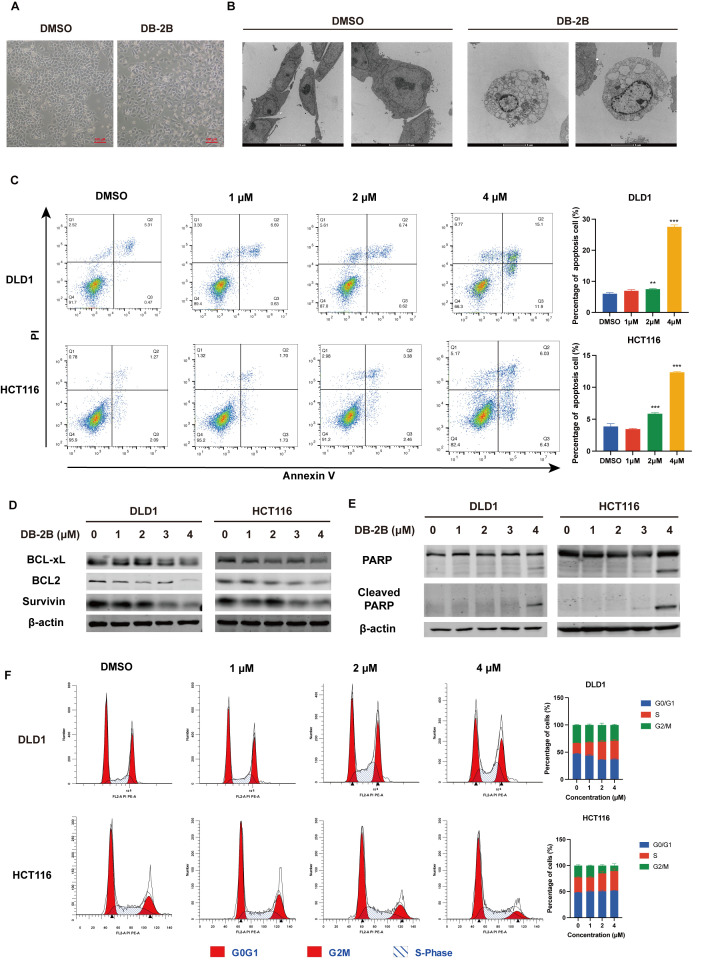
DB-2B induced apoptosis and cell cycle arrest in colorectal cancer cells. (**A**) Morphological changes in DLD1 cells following treatment with DB-2B under light microscopy. Scale bar = 10 μm. (**B**) Morphological changes in DLD1 cells following treatment with DB-2B under transmission electron microscopy. Scale bar = 5 μm. (**C**) Flow cytometry analysis of apoptosis rates in DLD1 and HCT116 cells following DB-2B treatment. (**D**) Protein levels of BCL2, BCL-xL, and Survivin in DLD1 and HCT116 cells treated with DB-2B. (**E**) Protein levels of PARP and cleaved PARP in DLD1 and HCT116 cells treated with DB-2B. (**F**) Cell cycle distribution of HCT116 and DLD1 cells treated with DB-2B. *p*-values were calculated using one-way ANOVA. ** *p* < 0.01, *** *p* < 0.001.

It was observed that DB-2B induced apoptotic morphological changes in CRC cells under light microscopy (Figure 4A). Further examination under transmission electron microscopy (TEM) showed chromatin margination, which is one of the typical early signs of apoptosis, confirming that DB-2B treatment induced apoptosis (Figure 4B). Flow cytometry analysis demonstrated increased apoptosis rates of 27.6% in DLD1 cells and 12.4% in HCT116 cells after treatment with 4 μM DB-2B for 24 h (Figure 4C). Western blot analysis showed downregulation of STAT3-regulated anti-apoptotic proteins BCL2, BCL-xL, and Survivin (Figure 4D). Increased cleavage of PARP, which is a hallmark of apoptosis, also confirmed that DLD1 and HCT116 cells underwent apoptosis following DB-2B treatment (Figure 4E). In addition to inducing apoptosis, DB-2B treatment disrupted cell cycle progression in both DLD1 and HCT116 cells, particularly impairing the transition from the S phase to the G2/M phase, as shown by flow cytometry analysis (Figure 4F). Collectively, these findings indicated that DB-2B inhibited the progression of CRC by promoting apoptosis and arresting cell cycle progression in abnormally proliferating CRC cells.

### 3.5. DB-2B Inhibited the Stemness of CRC Cells

**Figure 5:**
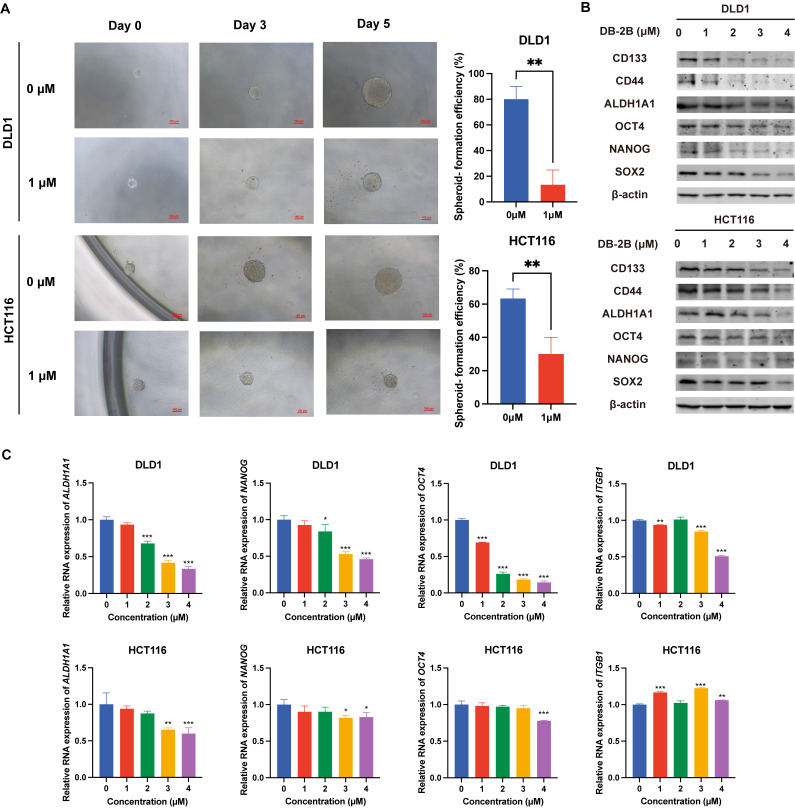
DB-2B inhibited cancer stemness in colorectal cancer cells. (**A**) Representative images of cellular spheroids derived from single DLD1 and HCT116 cells, and the spheroid-forming efficiency under DB-2B treatment. Scale bar = 100 μm. (**B**) Protein levels of stemness-associated genes in DLD1 and HCT116 cells following DB-2B treatment. (**C**) Relative mRNA expression levels of stemness-associated genes following DB-2B treatment. *p*-values were calculated using unpaired Student’s *t*-test or one-way ANOVA. * *p* < 0.05, ** *p* < 0.01, *** *p* < 0.001.

To investigate whether DB-2B treatment affected the maintenance of stemness of CRC cells, which has been shown to be associated with aberrant STAT3 activation, a spheroid formation assay was performed (Figure 5A). The result of the spheroid formation assay indicated that 1 μΜ DB-2B treatment of DLD1 and HCT116 cells reduced the spheroid size and significantly decreased the spheroid-forming efficiency. The mRNA expression levels of stemness-associated genes, including *ALDH1A1*, *NANOG*, and *OCT4*, were reduced in both DB-2B-treated DLD1 and HCT116 CRC cells (Figure 5C). In addition, ITGB1, a known promoter of stemness in CRC, was downregulated by DB-2B in DLD1 cells, whereas no significant downregulation was observed in HCT116 cells. This discrepancy may be attributed to the intrinsic heterogeneity between these two cell lines. Consistent with mRNA expression changes, the expression of stemness-associated genes was also validated at the protein level. It was found that the expression levels of CD133, CD44, ALDH1A1, OCT4, NANOG, and SOX2 decreased after 48 h of DB-2B treatment (Figure 5B). The reduction in the protein level of stemness-associated markers induced by DB-2B was more pronounced than that induced by LY17 at the same concentration, suggesting an enhanced inhibitory effect of DB-2B on stemness compared with LY17 (Figure S5). Taken together, our findings suggested that DB-2B suppressed the expression of stemness-related genes including *ALDH1A1*, *OCT4*, and *NANOG*, thereby inhibiting the stemness of CRC cells.

### 3.6. DB-2B Exhibited Favorable Pharmacokinetic Properties and Oral Bioavailability

**Figure 6:**
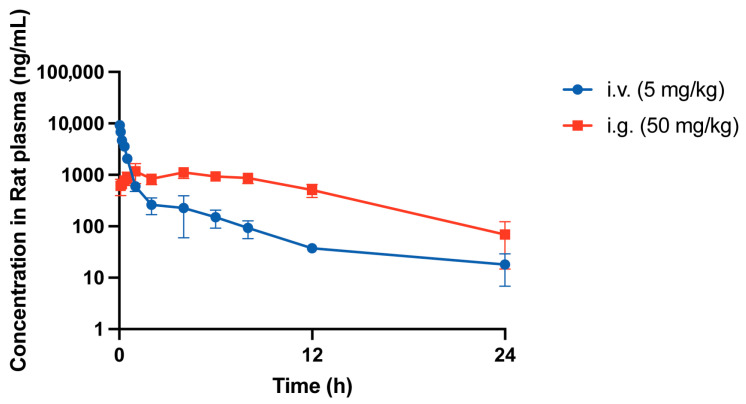
Plasma concentration–time curves of DB-2B following intravenous administration (5 mg/kg) and oral gavage (50 mg/kg) in rats. DB-2B was administered to rats via intravenous injection (5 mg/kg, n = 3) or oral gavage (50 mg/kg, n = 4). Plasma drug concentrations were measured at the indicated time points.

To determine whether DB-2B exhibited favorable pharmacokinetic properties and favorable oral bioavailability, we evaluated its pharmacokinetic profile in vivo. The plasma concentration curves following intravenous (5 mg/kg) and oral (50 mg/kg) administration demonstrated stable pharmacokinetic properties (Table 1, Figure 6). Following oral administration, DB-2B exhibited high systemic exposure in rats, with a C_max_ of 1338.36 ± 257.85 ng/mL, an AUC_(0–∞)_ of 14,454.64 ± 1604.55 h·ng/mL, and a bioavailability of 26.37%. In summary, DB-2B demonstrates favorable in vivo pharmacokinetic properties and warrants further investigation as a potential therapeutic agent for CRC treatment.

### 3.7. Oral Administration of DB-2B Inhibited CRC Progression In Vivo

**Figure 7:**
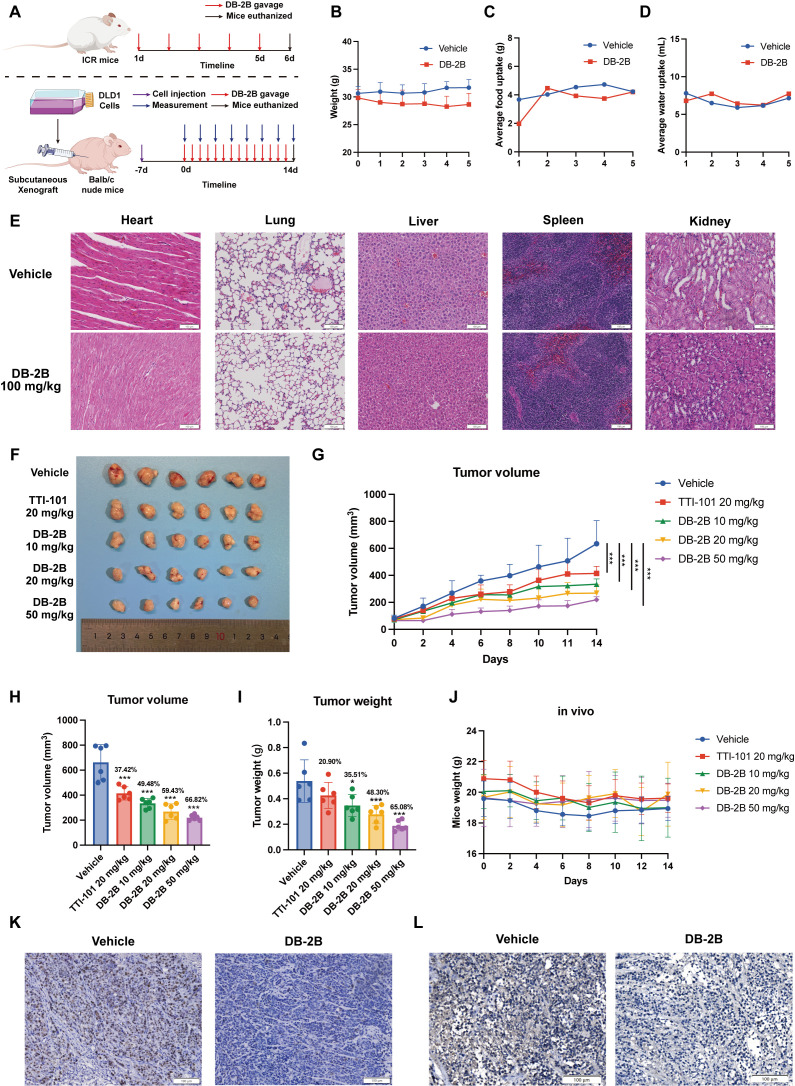
DB-2B inhibited colorectal cancer (CRC) progression in vivo. (**A**) Schematic representation of the in vivo models used in this study, including the drug toxicity assay (top) and the subcutaneous xenograft tumor model (bottom). (**B**–**D**) Body weight, food intake, and water intake of mice during 100 mg/kg/d DB-2B treatment (n = 3 per group). (**E**) Representative images of hematoxylin and eosin (H&E) staining of the heart, lung, liver, spleen, and kidney from mice treated with DB-2B (100 mg/kg, once daily for 5 days). Scale bar = 100 μm. (**F**) Tumor growth following DB-2B treatment (n = 6 per group). (**G**–**I**) Growth rate, tumor volume, and tumor weight following DB-2B treatment and vehicle. (**J**) Body weight of mice during treatment. (**K**) Representative images of immunohistochemical (IHC) staining of Ki67 in tumors following DB-2B administration. Scale bar = 100 μm. (**L**) Representative images of IHC staining of phosphorylated STAT3 in tumors after DB-2B administration. Scale bar = 100 μm. *p*-values were calculated using one-way ANOVA. * *p* < 0.05, *** *p* < 0.001.

Having confirmed that DB-2B exhibited favorable pharmacokinetic properties and oral bioavailability, we further evaluated its potential as an oral anticancer drug for CRC. Mice administered 100 mg/kg DB-2B via daily gavage for 5 consecutive days showed no significant changes in food or water intake, and only a slight decrease in body weight (Figure 7A–D). H&E staining of the heart, lung, liver, spleen, and kidney showed no notable histopathological alterations following high-dose oral administration of DB-2B. These results suggested that DB-2B exhibited a favorable safety profile in vivo (Figure 7E).

The antitumor efficacy of DB-2B was subsequently evaluated using a CRC xenograft model (Figure 7A,F). TTI-101, a STAT3 inhibitor previously demonstrated to be effective in preclinical studies and which had completed a Phase I clinical trial, was used as a positive control [36]. Tumor volumes were monitored throughout the treatment period, during which mice received oral gavage of DB-2B at 10 mg/kg, 20 mg/kg, and 50 mg/kg for 14 consecutive days. The growth rate of tumors was significantly inhibited by DB-2B treatment (Figure 7G). By the end of the 14-day treatment period, tumor volumes were reduced by 49.48%, 59.43%, and 66.82% in the 10 mg/kg, 20 mg/kg, and 50 mg/kg groups, respectively (Figure 7H). Correspondingly, tumor weights decreased by 35.51%, 48.30%, and 65.08% in the respective dosage groups (Figure 7I). No significant weight loss was observed in the treated mice compared with the vehicle control group, and H&E staining revealed no notable histopathological changes in major organs, further supporting the in vivo safety of DB-2B (Figure 7J and Figure S6). A reduction in Ki-67-positive cells in the DB-2B-treated group further demonstrated that DB-2B suppressed CRC cell proliferation in vivo (Figure 7K). IHC staining of xenograft tumors confirmed that STAT3 phosphorylation was inhibited by DB-2B in vivo (Figure 7L). In conclusion, DB-2B is orally effective in inhibiting CRC progression by targeting STAT3 activation in vivo.

## 4. Discussion

Research on the JAK/STAT signaling pathway has spanned more than three decades [1]. Shortly after its discovery, the critical role of the JAK/STAT pathway in tumor progression was recognized, prompting further investigation into its functions within the tumor microenvironment (TME). Consequently, the JAK/STAT signaling pathway has remained a prominent target for molecular targeted therapies due to its indispensable role in cancer progression and immune regulation [37]. Compared with the rapid development of JAK inhibitors, with numerous JAK inhibitors now approved for the treatment of various indications, the development of STAT3 inhibitors has encountered substantial challenges. For example, the stemness inhibitor Napabucasin has completed phase III clinical trials in CRC, pancreatic cancer, and GC, as well as several phase I/II trials in other tumor types. However, Napabucasin was found to confer no significant benefit to overall survival (OS) or progression-free survival (PFS) in these phase III clinical trials [20,21,22,23,24,25,26]. Only the CO.23 clinical trial reported improved survival in patients with pSTAT3-positive tumors [23]. TTI-101, another inhibitor targeting the SH2 domain of STAT3, exhibited a favorable safety profile in a Phase I clinical trial [36]. However, during phase I clinical trials, researchers observed that TTI-101 exhibited greater efficacy in hepatocellular carcinoma (HCC) than that observed in other advanced solid tumors. As a result, the focus of the subsequent Phase II clinical trials was narrowed from a broad range of solid tumors to HCC [36,38]. To date, no STAT3 inhibitor has been validated in clinical trials and approved for the treatment of CRC [39].

One of the main reasons for the challenges of developing STAT3 inhibitors is that the molecular structure of STAT3 is relatively flat and lacks a deep pocket that could accommodate effective drug binding, rendering it an “undruggable” target. Many compounds used to target STAT3 activation are derived from natural products and their derivatives; however, most of them lack specificity and act on multiple signaling pathways [40,41]. Targeting specific functional domains has emerged as a promising strategy to enhance the specificity of STAT3 inhibitors. Among these domains, the SH2 domain has become an ideal target, as it serves as the primary site responsible for STAT3 activation and dimerization [2,4]. Phosphopeptide-based compounds targeting the SH2 domain of STAT3 exhibit high binding affinity and specificity, but show poor cellular activity due to limited membrane permeability [42]. In addition, phosphopeptide-based proteolysis-targeting chimeras (PROTACs) targeting STAT3 have emerged as a major research focus in recent years [43,44]. These agents do not inhibit STAT3 activity directly but instead downregulate the levels of STAT3 protein, thereby overcoming the limited cellular activity observed with some STAT3 inhibitors. However, one of the major limitations of phosphopeptide PROTACs is their limited oral bioavailability. Overall, many STAT3 inhibitors currently in development, despite showing promising results in preclinical studies, struggle to advance into clinical use due to poor druggability.

Another target once regarded as “undruggable,” KRAS, has also faced repeated setbacks in inhibitor development [45]. The emergence of the KRASG12C inhibitor ARS-1620 and AMG510 marked a turning point, ushering in a new era of rapid therapeutic development [45,46,47]. Since then, multiple inhibitors targeting KRAS^G12C^, KRAS^G12D^, and KRAS^G12V^ have entered clinical trials, with some already approved for clinical use, offering new treatment options for cancer patients [45,48]. The success of KRAS-targeted therapies not only inspires STAT3 drug development but also offers valuable insights. With a deepening understanding of the structure and function of STAT3, coupled with advances in medicinal chemistry and structural optimization, STAT3 holds promise as a druggable target that could ultimately benefit clinical patients.

An optimal strategy for STAT3 inhibition remains elusive. Thus, it is necessary to strive for a balance between activity, safety, targeting, and druggability. Therefore, the development of more effective, safer, more specific, and better orally bioavailable STAT3-targeted inhibitors remains an important research direction. The novel STAT3 inhibitor DB-2B was designed based on the structure of the SH2 domain of the STAT3 protein, as well as our previously developed STAT3 inhibitor LY17, aiming to optimize the potentially reactive chemical structure of LY17 while maintaining specificity for STAT3 [27]. The binding of DB-2B to the SH2 domain was confirmed by molecular docking analysis. By occupying the SH2 docking site, DB-2B interferes with STAT3 activation and nuclear translocation. This inhibition subsequently affects the expression of genes downstream of the transcription factor STAT3, such as *MYC*, *BIRC5*, and *BCL2*, thereby impairing tumor proliferation, metastasis, and biological functions including stemness. Notably, optimization of the potentially reactive chemical structure of LY17 conferred the compound with enhanced cytotoxicity and superior ability to inhibit stemness markers. DB-2B showed a negligible effect on the activation of upstream JAK or on other members of the STAT family including STAT1 and STAT5. Furthermore, DB-2B could effectively inhibit tumor growth in CRC subcutaneous xenograft models in vivo with favorable biosafety, demonstrating its potential as an anticancer drug for CRC.

In addition to the development of more effective STAT3 inhibitors, it is important to acknowledge that the human TME is considerably more complex and heterogeneous than that of xenograft models. STAT3 is expressed in multiple cell types within the TME, particularly in immune cells, and promotes the formation of immunologically “cold” tumors [49,50,51,52]. In pancreatic cancer, it has also been reported that STAT3 inhibition can result in the transformation from differentiated CAF to mesenchymal stem cell CAF, enhancing the efficacy of PD-1 immunotherapy [53,54,55]. Therefore, exploring more effective combination strategies involving STAT3 inhibitors and other treatment modalities represents a highly promising research direction for improving therapeutic outcomes. In this study, only cell line-derived xenograft models were used, which lack the capacity to fully simulate the TME found in syngeneic mouse models or genetically engineered models. Future studies incorporating diverse preclinical models and both sexes are warranted to validate the clinical potential of DB-2B, particularly in combination with chemotherapeutic agents or immunotherapies. Furthermore, although our preliminary short-term toxicity assessment indicated a favorable safety profile for DB-2B, the limited duration of evaluation restricts the detection of potential delayed or cumulative toxicities. Accordingly, more comprehensive safety studies are required, including cytotoxicity evaluations in normal cells and long-term in vivo toxicity assessments under high-dose or chronic administration conditions, to further support the clinical translation of DB-2B.

## 5. Conclusions

In conclusion, by optimizing the potentially reactive structure of our previously developed STAT3 inhibitor LY17, we have designed and synthesized a novel STAT3 inhibitor DB-2B using a structure-based drug design strategy to specifically target the SH2 docking site of STAT3. SPR, ITC, and CETSA assays confirmed that DB-2B bound to STAT3 with high affinity, in line with our computational analysis, indicating favorable target specificity. In vitro, DB-2B exhibited anticancer function by inhibiting the growth, proliferation, migration and invasion of CRC cells. As a STAT3 inhibitor, DB-2B induced apoptosis in CRC cells, thereby reducing viability. It also arrested cell-cycle progression by impairing entry into the G2/M phase, ultimately preventing the completion of mitosis. Furthermore, DB-2B suppressed CRC stemness, highlighting its potential as a cancer stemness inhibitor. In vivo, orally administered DB-2B demonstrated favorable and predictable pharmacokinetic properties and significantly suppressed CRC progression in animal models. Collectively, our findings suggest that the rationally designed and synthesized compound DB-2B exhibits promising druggability and may serve as an oral STAT3 inhibitor for the treatment of CRC.

## Figures and Tables

**Table 1:** Main pharmacokinetic parameters of DB-2B in rats.

Parameter	Units	i.v. (5 mg/kg)	i.g. (50 mg/kg)
C_0_ (C_max_)	ng/mL	11,053.99 ± 2803.28	1338.36 ± 257.85
T_max_	h	/	3.00 ± 2.45
AUC_(0-t)_	h·ng/mL	5278.69 ± 889.09	14,233.60 ± 1608.64
AUC_(0-∞)_	h·ng/mL	5481.36 ± 886.59	14,454.64 ± 1604.55
t_1/2_	h	7.13 ± 1.38	4.20 ± 1.17
V_d_(V/F)	L/kg	9.56 ± 2.83	21.16 ± 6.06
CL(CL/F)	L/h/kg	0.93 ± 0.14	3.49 ± 0.38
MRT_(0-∞)_	h	4.62 ± 1.36	8.38 ± 1.64
F (%)	%	/	26.37

## Data Availability

The original contributions presented in this study are included in the article. Further inquiries can be directed to the corresponding author.

## Supplementary Materials

The following supporting information can be downloaded at: https://www.mdpi.com/article/10.3390/biom16050752/s1, Figure S1. ^1^H NMR spectrum of DB-2B (300 MHz, DMSO-d6); Figure S2. ^13^C NMR spectrum of DB-2B (75 MHz, DMSO-d6); Figure S3. HPLC spectrum of DB-2B (75 MHz, DMSO-d6); Figure S4. HR-ESI-MS spectrum of DB-2B; Figure S5. Effect of LY17 on cell viability and stemness-associated protein expression in colorectal cancer cells; Figure S6. DB-2B exhibits in vivo safety in mice; Table S1. List of antibodies and specifications; Table S2. List of primer sequences used for qPCR assay.
