# Supplementary material for: DB-2B, a Novel and Selective STAT3 Inhibitor Inhibits Colorectal Cancer Progression In Vitro and In Vivo
Source: Biomolecules. 2026 May 20;16(5):752. doi: 10.3390/biom16050752 (PMC13204227; doi:10.3390/biom16050752)
Supplement: Supplementary file 1 [file biomolecules-16-00752-s001.zip › Supplemantary Materials.pdf]

Supplementary Materials

Supplementary Methods

Synthesis of DB-2B

1. Preparation of *naphthalene-1-sulphonyl chloride* (2a)

To a solution of sodium-naphthalenesulfonate (50 g, 233.4 mmol) in 200 mL MeCN/tetramethylene sulfone (1:1) was added 70 mL phosphorus oxychloride at 0 °C, and then the solution was heated at 70 °C for 1 hour and cooled to room temperature. 300 mL water was added to the mixture, after which white solids precipitated and was stirred at room temperature for 1 hour and filtered. The solids were washed with water and then dried under vacuum to afford the title compound 2a (44.5 g, 84.1%).

^{1}H NMR (400MHz, DMSO-d_6) δ 8.65 (d, *J*=8.3 Hz, 1H, Ar-H), 8.20 (d, *J*=8.2 Hz, 1H, ArH), 8.17 – 8.06 (m, 2H, Ar-H), 7.75 – 7.69 (m, 1H, Ar-H), 7.69 – 7.68 (m, 1H, Ar-H), 7.67 (d, *J*=3.9 Hz, 2H, NH_2), 7.63 (d, *J*=7.9 Hz, 1H, Ar-H).

2. Preparation of *naphthalene-1-sulfonamide* (3a)

To a solution of 2a (15 g, 66.2 mmol) in 40 mL acetone at -10 °C was added 14 mL ammonium hydroxide dropwise, after which the mixture was stirred at room temperature for 2 hours. Then the solution was concentrated under reduced pressure and 200 mL water was added to redissolve the residue. The aqueous solution was extracted with EA (3×100 mL). Then the organic phase was combined and washed with water and saturated NaCl solution

and dried over Na_2SO_4. The solvent was removed under reduced pressure to afford the title compound 3a (12.1 g, 88.2%).

^{1}H NMR (300MHz, DMSO-d_6) δ 8.45 (d, J=9.2 Hz, 1H, Ar-H), 8.28 (d, J=8.9 Hz, 1H, ArH), 8.06 (t, J=7.8 Hz, 1H, Ar-H), 7.38 (s, 2H, NH_2), 7.23 – 7.10 (m, 2H, Ar-H).

3. Preparation of *5,8-dioxo-5,8-dihydronaphthalene-1-sulfonamide* (4a)

To a solution of $Ce(SO_4)_2$ (112 g, 337 mmol) in 150 mL 2M H_2SO_4 was added a mixture of 3a (7 g, 33.7 mmol) in 140 mL MeCN at 70 °C. The solution was stirred at 70 °C for 30 minutes, and then poured into 100 mL ice water with an excess amount of NaCl added. Then the mixture was filtered and the filtrate was collected and extracted with DCM. After combining and washing the organic layer with water, the solution was dried over Na_2SO_4. The solvent was removed under reduced pressure to afford the title compound 4a (3.5 g) which can be used in next step without further purification.

4. Preparation of *3-(ethylamino)-5H-naphtho[1,8-cd]isothiazol-5-one 1,1-dioxide* (5a)

To a mixture of 4a (3.5 g, 14.7 mmol) in 40 mL MeOH was added ethylamine (1.3 g, 29.5 mmol) dropwise at room temperature. Then the reaction was stirred at room temperature for 2 hours. The solvent was removed under reduced pressure. Then column chromatography on SiO_2 (petroleum ether/EA=2:1) afforded the title compound 5a (1.54 g, 39.8%).

5. Preparation of *3-(3-(ethylamino)-1,1-dioxido-5-oxo-5H-naphtho[1,8-cd]isothiazol-4-yl)benzenaminium chloride* (DB-2B)

A solution of N-Boc-3-aminophenylboronic acid (1.17 g, 8.58 mmol), $Mn(OAc)_2$ (2.97 g, 17.16 mmol), 5a (1.5 g, 5.72 mmol) in 10 mL DMSO was heated at 105 °C overnight. Then the reaction was cooled to room temperature and was added 25 mL water. The mixture was extracted with EA and the organic layer was combined and dried with Na_2SO_4. Then the solvent was removed under reduced pressure and purified with column chromatography on SiO_2 to afford red solids. The solids were redissolved in 10 mL DCM and bubbled with HCl at 0 °C for 3 hours, after which the solution was filtered to obtain the crude product which was washed with cooled DCM to afford the title compound DB-2B (0.45 g, 20.2%).

^{1}H NMR (300 MHz, DMSO-d_6) δ 10.16 (s, 2H, NH_2), 8.39 (dd, J=7.1, 1.4 Hz, 1H, Ar-H), 8.19 – 7.88 (m, 2H, Ar-H), 7.52 (t, J=7.8 Hz, 1H, NH), 7.44 – 7.09 (m, 4H, Ar-H), 2.86 (q, =6.8 Hz, 2H, CH_2CH_3), 0.92 (t, J=7.1 Hz, 3H, CH_2CH_3). ^{13}C NMR (75 MHz, DMSO-d_6) δ 179.45, 162.39, 143.27, 136.84, 136.37, 135.72, 133.30, 130.31, 129.71, 129.50, 128.55, 127.78, 125.40, 122.15, 117.79, 15.40; HRMS (ESI) calcd for $C_{18}H_{15}N_3O_3S$ $[M+H]^+$ 354.0956, found 354.0956.

RNA isolation and Real-time PCR

Total RNA was extracted using the E.Z.N.A.® Total RNA Kit I (Omega Bio-tek, Norcross, GA, USA). RNA concentration and purity were measured using a NanoDrop spectrophotometer (Thermo

Fisher Scientific, Waltham, MA, USA). Complementary DNA was transcribed using the HiScript II kit (Vazyme, Nanjing, China) and subjected to qPCR analysis using ChamQ

Universal SYBR Master Mix (Vazyme) on a LightCycler® 480 II (Roche, Basel, Switzerland). Primer sequences used for qPCR were listed in Table S2.

Protein extraction and Western blot analysis

Whole cell lysates were prepared using RIPA lysis buffer (Beyotime, Shanghai, China) supplemented with PMSF (Servicebio, Wuhan, China) and phosphatase inhibitors (Beyotime, Shanghai, China). BCA assay was performed to determine the concentration of protein using the Pierce™ BCA Protein Assay Kit (Thermo Fisher Scientific, Waltham, MA, USA). The protein lysates were mixed with sample loading buffer and boiled for 5 minutes at 100 °C in a dry bath with metal heating block.

Proteins were separated via SDS-PAGE and transferred to nitrocellulose membrane (MilliporeSigma, Burlington, MA, USA). 5% skim milk was used to block nonspecific binding.

Following overnight incubation with primary antibodies at 4 °C, the membranes were incubated with IRDye® 680 LT secondary antibodies (LI-COR Biosciences, Lincoln, NE, USA) for 1 hour. Subsequently, fluorescent signals were visualized using the Odyssey DLx Infrared Imaging System (LI-COR Biosciences, Lincoln, NE, USA).

H&E staining and immunohistochemistry

Sections of 4 μm thickness were cut from formalin-fixed, paraffin-embedded (FFPE) blocks and subsequently transferred onto adhesive slides (Citotest, Jiangsu, China). Slides

were deparaffinized by immersion in xylene, followed by rehydration through a graded ethanol series (100%, 95%, 75%) and a final wash in distilled water.

For Hematoxylin and Eosin (H&E) staining, rehydrated sections were stained with hematoxylin for 10 minutes, rinsed under running tap water, differentiated in 1% acid ethanol, and blued in running tap water for 30 minutes. Slides were counterstained with eosin for 10 seconds, dehydrated through graded alcohols, cleared in xylene, and mounted with neutral resin. Histological features were evaluated using Olympus BX53 light microscopy (Olympus Corporation, Tokyo, Japan).

For Immunohistochemistry (IHC), antigen retrieval was performed in a pressure cooker using 0.01 M citrate buffer (pH 6.0) for 3 minutes at full pressure, followed by natural cooling. Slides were treated with 3% hydrogen peroxide in methanol for 15 minutes, washed, and then blocked in 5% NBS for 30 minutes prior to overnight incubation with primary antibodies at 4 °C. Detection was carried out using HRP-conjugated secondary antibodies and a DAB substrate kit (ZSGB-BIO, Beijing, China), followed by hematoxylin counterstaining, dehydration, and mounting.

Images were captured and analyzed using a digital slide scanner (Olympus, Tokyo, Japan) or a light microscope.

Supplementary Figures

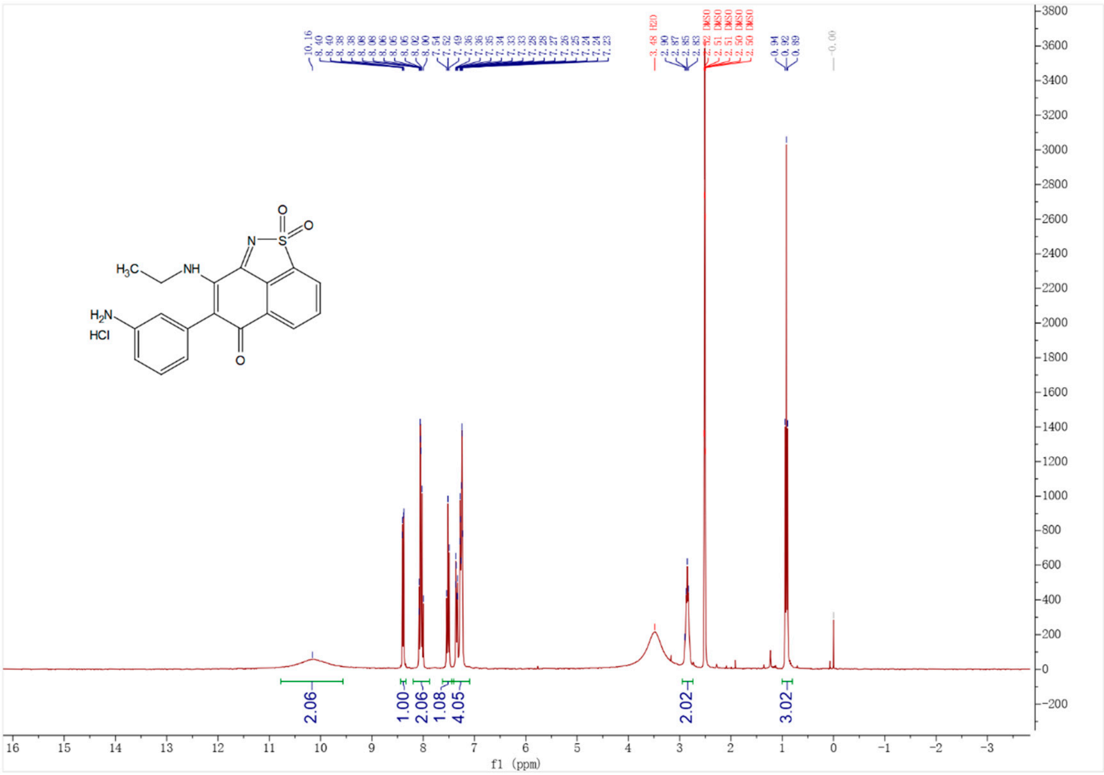

Figure S1. **^{1}H NMR spectrum of DB-2B (300 MHz, DMSO-d_6).**

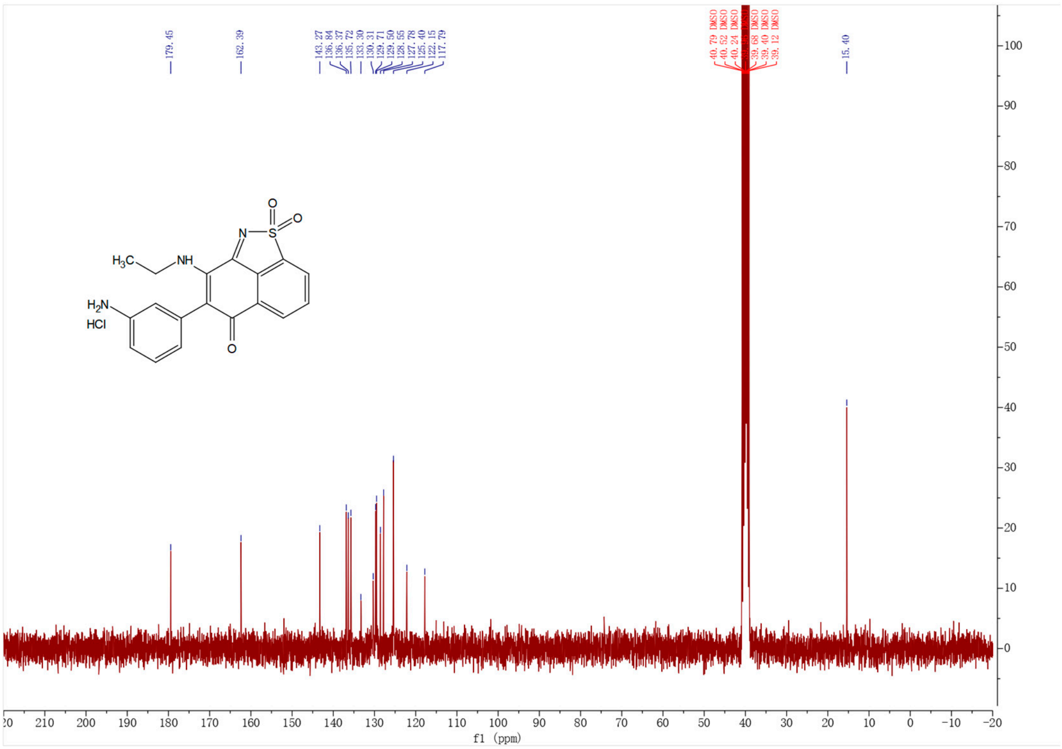

Figure S2. ^{13}**C NMR spectrum of DB-2B (75 MHz, DMSO-d$_6$).**

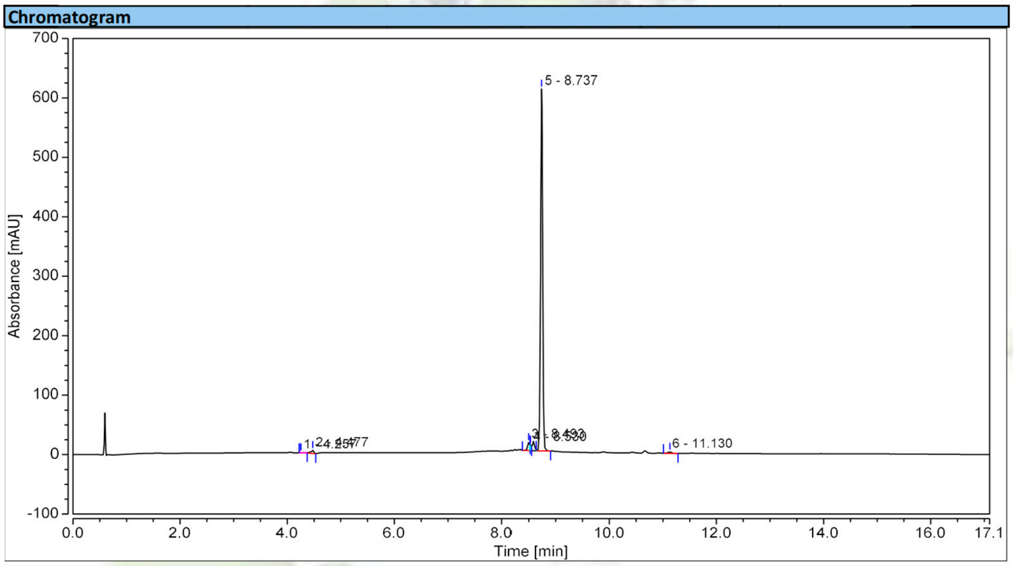

Integration Results

No.	Peak Name	Retention Time min	Area mAU*min	Height mAU	Relative Area %	Relative Height %	Amount n.a.
1		4.257	0.005	0.045	0.02	0.01	n.a.
2		4.477	0.393	5.035	1.38	0.79	n.a.
3		8.493	0.641	13.633	2.24	2.13	n.a.
4		8.530	0.258	9.550	0.90	1.49	n.a.
5		8.737	27.112	608.700	94.86	95.22	n.a.
6		11.130	0.173	2.296	0.61	0.36	n.a.
**Total:**			**28.581**	**639.258**	**100.00**	**100.00**	

Figure S3. HPLC spectrum of DB-2B.

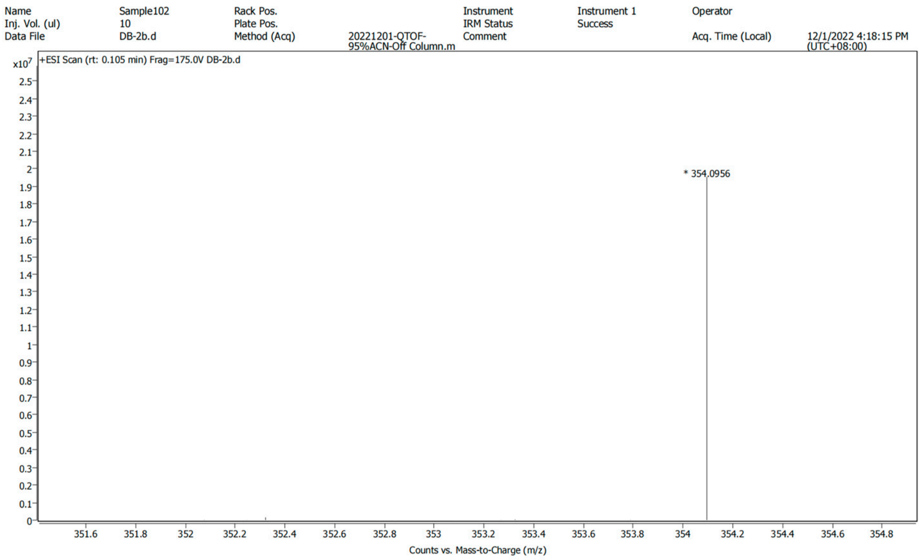

Figure S4. HR-ESI-MS spectrum of DB-2B.

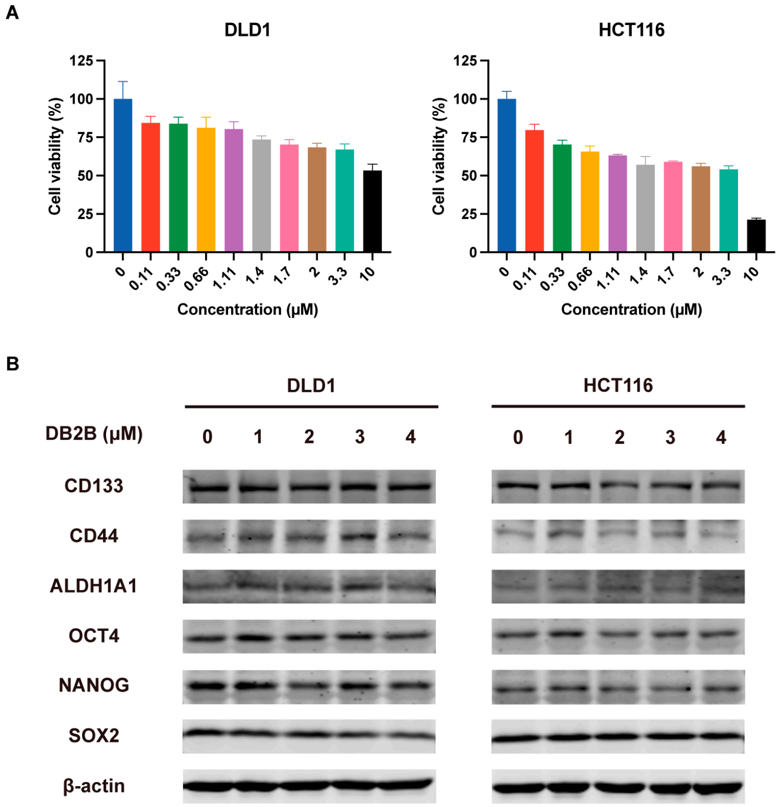

Figure S5. Effect of LY17 on cell viability and stemness-associated protein expression in colorectal cancer cells. (A) Cell viability of DLD1 and HCT116 cells following 24-hour treatment with increasing concentrations of LY17 (IC50: 6.677 μM for DLD1; 2.511 μM for HCT116). (B) Protein level of stemness-associated genes in DLD1 and HCT116 cells following LY17 treatment.

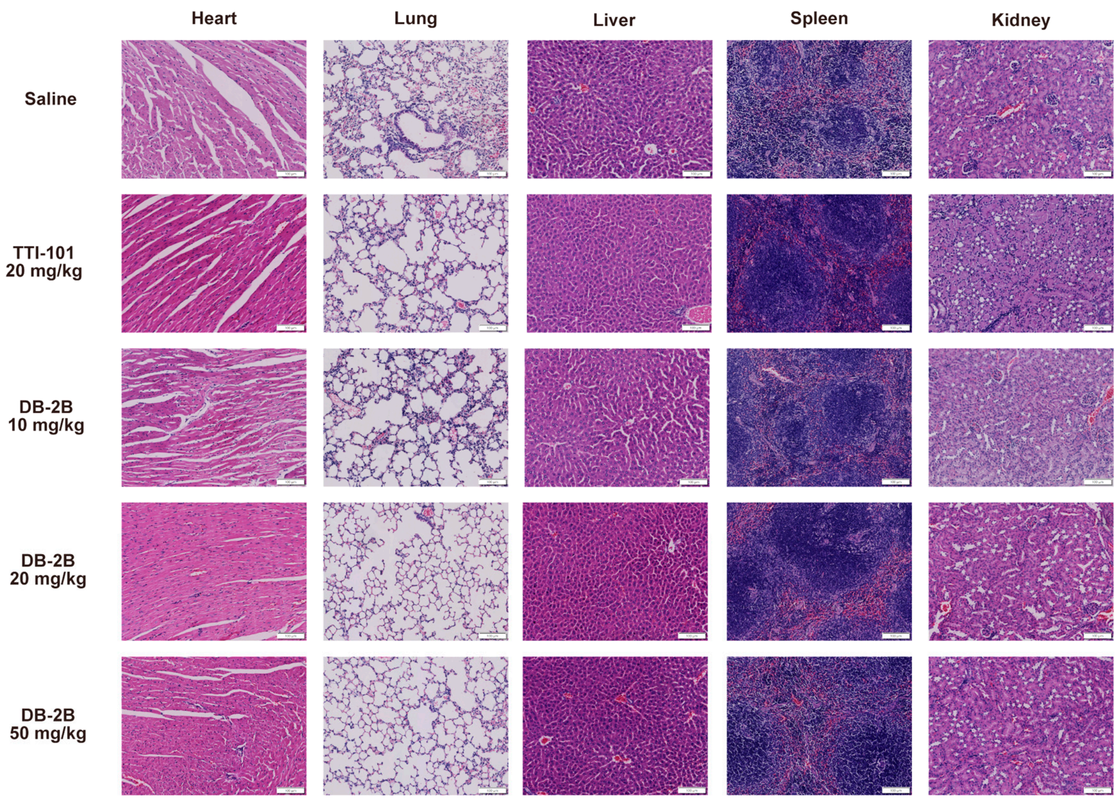

Figure S6. DB-2B exhibits in vivo safety in mice. Representative images of Hematoxylin and eosin (H&E) staining of the heart, lungs, liver, spleen, and kidneys in mice treated with DB-2B (10 mg/kg, 20 mg/kg and 50 mg/kg, once daily for 14 days) or TTI-101 (20 mg/kg, once daily for 14 days).

Supplementary Tables

Table S1. List of antibodies and specifications.

Target	Company	Catalog number	Application	Dilution concentration
STAT3	Cell Signaling Technology	9139	WB	1:1000
			IF	1:1000
p-STAT3 (Tyr705)	Cell Signaling Technology	9145	WB	1:2000
			IHC	1:100
p-STAT3 (Ser727)	HUABIO	ET1607-39	WB	1:1000
STAT1	Cell Signaling Technology	14994	WB	1:1000
p-STAT1	Cell Signaling Technology	9167	WB	1:1000
STAT5	HUABIO	ET1701-45	WB	1:1000
p-STAT5	HUABIO	ET1610-48	WB	1:1000
JAK1	HUABIO	ET1705-84	WB	1:1000
p-JAK1	abmart	TP56310	WB	1:1000
JAK2	HUABIO	ET1607-35	WB	1:1000
p-JAK2	abmart	T56570	WB	1:1000
BCL-xL	Cell Signaling Technology	2764	WB	1:1000

BCL-2	Cell Signaling Technology	15071	WB	1:1000
Survivin	Cell Signaling Technology	2808	WB	1:1000
PARP	Cell Signaling Technology	9532	WB	1:1000
Cleaved PARP	Cell Signaling Technology	5625	WB	1:1000
CD133	HUABIO	HA601024	WB	1:1000
CD44	Cell Signaling Technology	3570	WB	1:1000
ALDH1A1	Proteintech	15910-1-AP	WB	1:5000
OCT4	HUABIO	HA601207	WB	1:1000
NANOG	HUABIO	ET1610-2	WB	1:1000
SOX2	HUABIO	R1106-1	WB	1:1000
Ki67	Abcam	ab15580	IHC	1:1000

Table S2. List of primer sequences used for qPCR assay.

Genes	Forward primer	Reverse primer
ACTB	GTCATTCCAAATATGAGATGCGT	GCTATCACCTCCCCTGTGTG
BIRC5	GCCTGGCAGCCCTTTCTCAAG	CAAGTCTGGCTCGTTCTCAGTGG

MYC	AATGAAAAGGCCCCCAAGGTAGTTATCC	GTCGTTTCCGCAACAAGTCCTCTTC
ALDH1A1	GCCAGTGTTGTATAGCCGCA	TCCACATTCCAGTTTGGCCC
NANOG	TGTCTCTCCTCTTCCTTCCTCCATG	TGGGTTGTTTGCCTTTGGGACTG
OCT4	GATGTGGTCCGAGTGTGGTT	AGAGTGGTGACGGAGACAGG
ITGB1	TGAAGGGCGTGTTGGTAGAC	AGACACCACACTCGCAGATG
