# Supplementary figures and images for: DB-2B, a Novel and Selective STAT3 Inhibitor Inhibits Colorectal Cancer Progression In Vitro and In Vivo
Source: Biomolecules. 2026 May 20;16(5):752. doi: 10.3390/biom16050752 (PMC13204227; doi:10.3390/biom16050752)

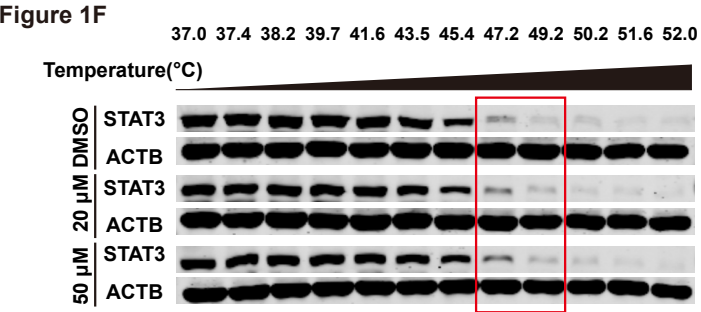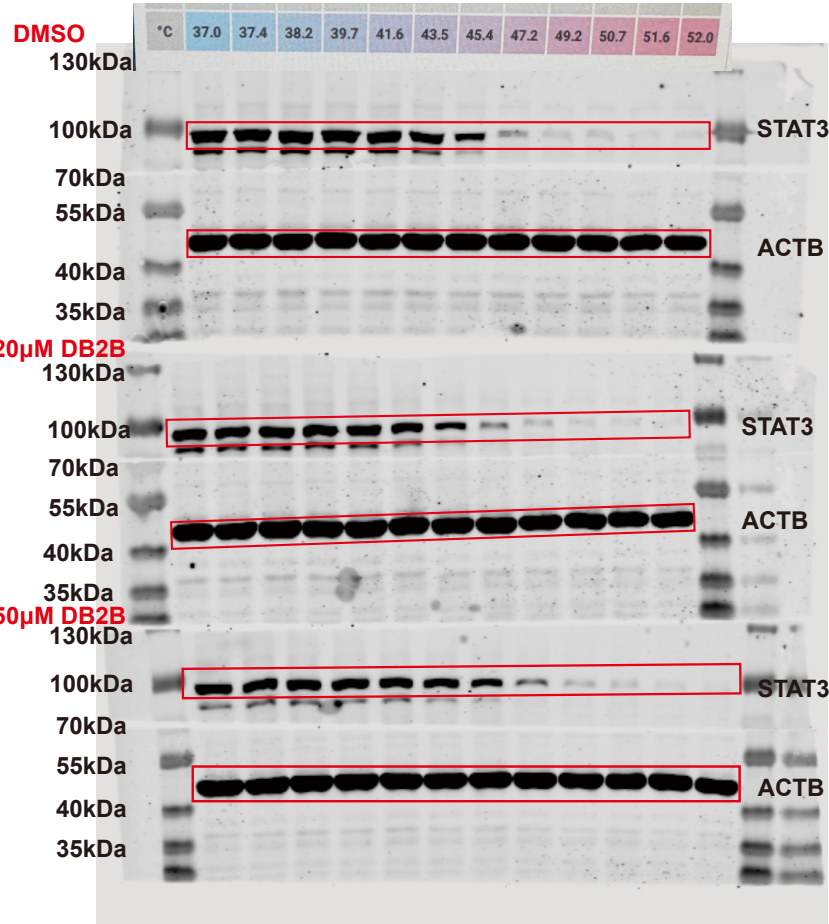

**Figure 2A**

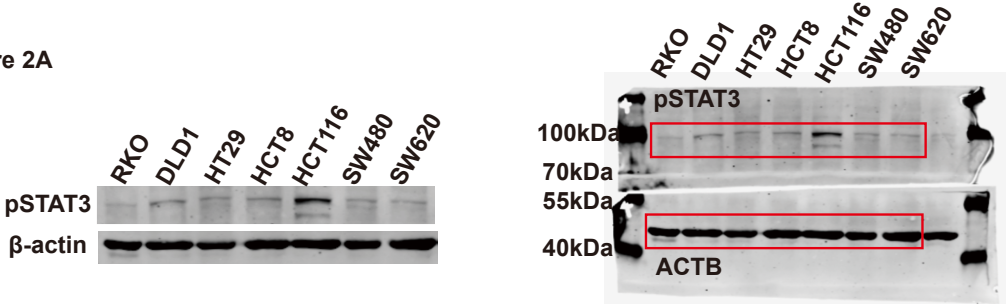

### Figure 2C

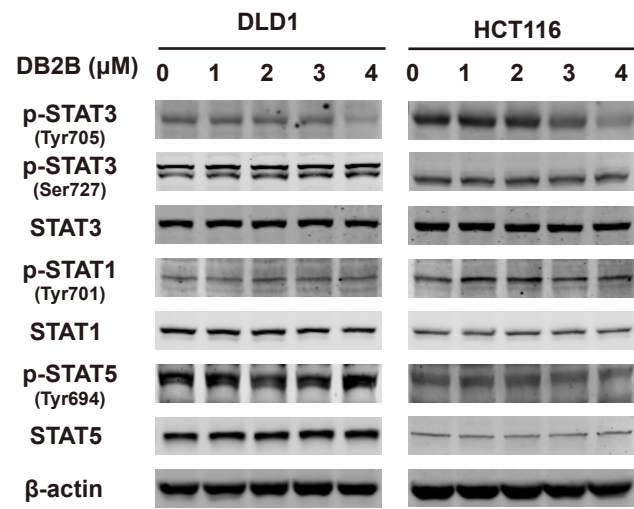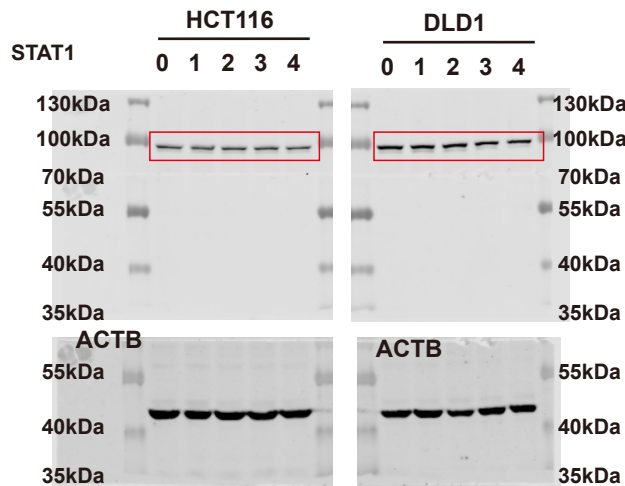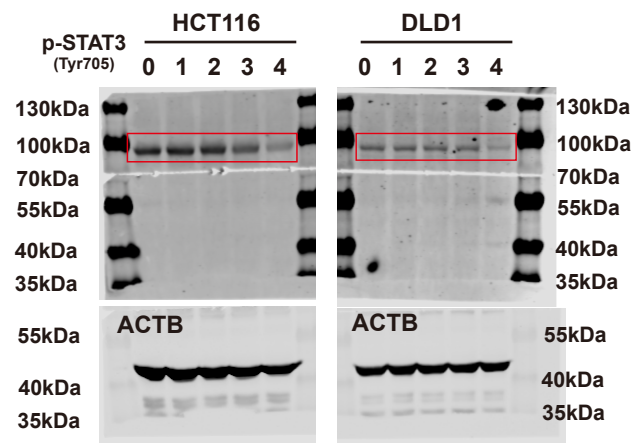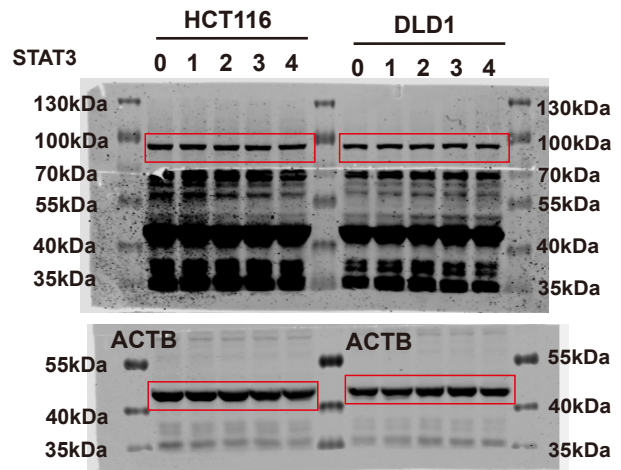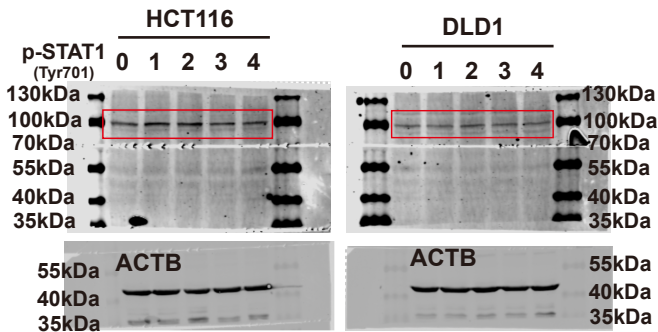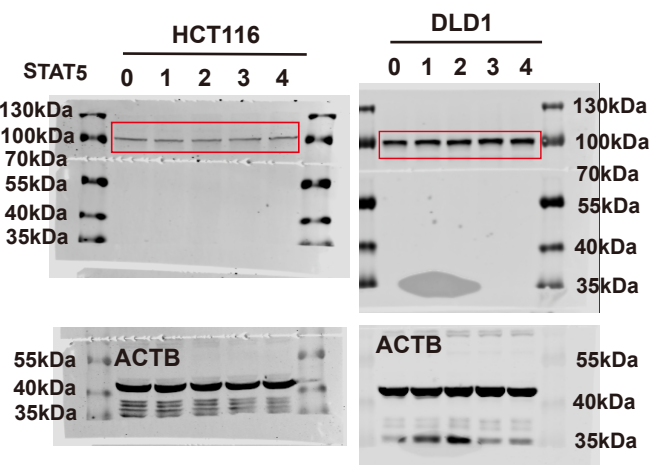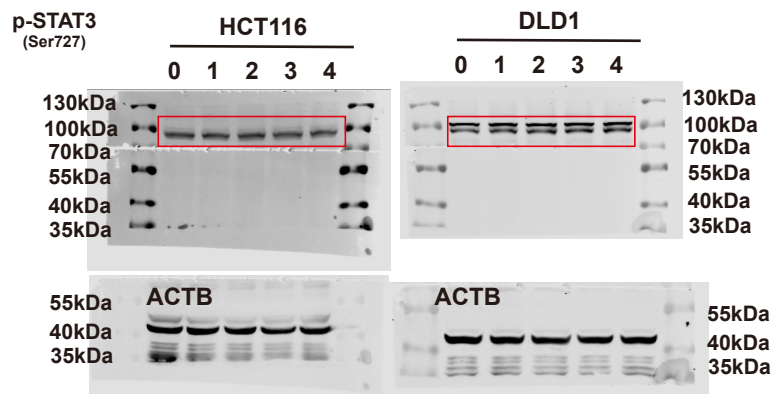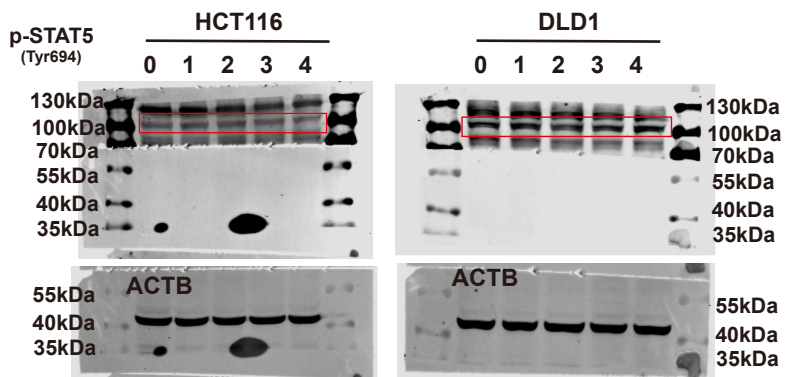

### Figure 2D

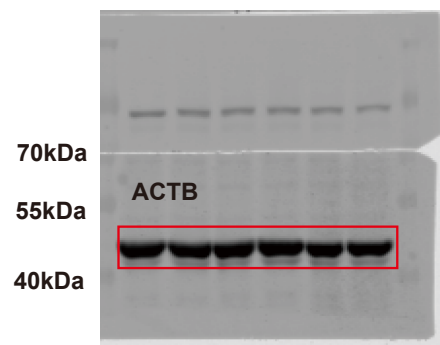

Figure 2E

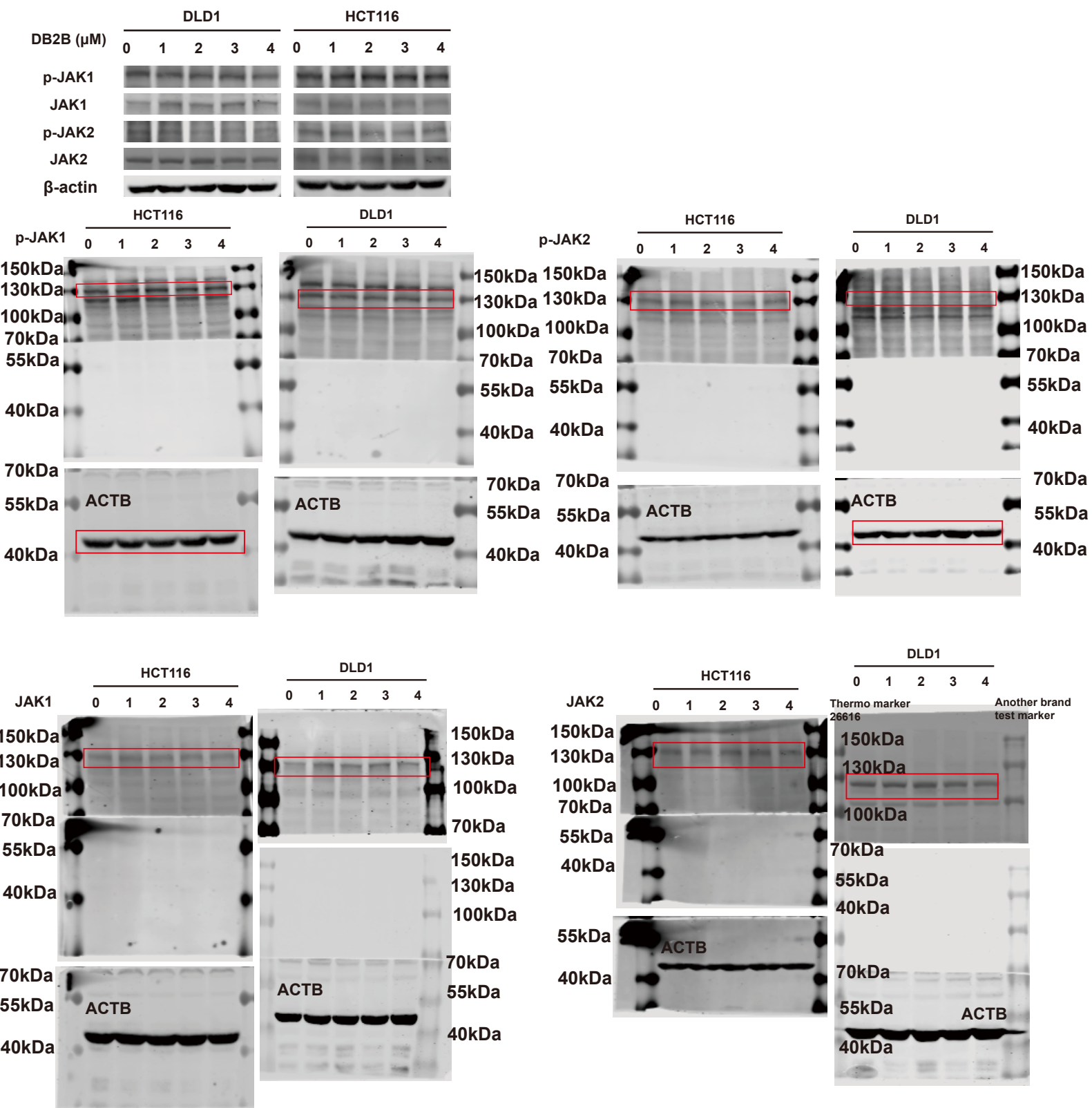

Figure 4D

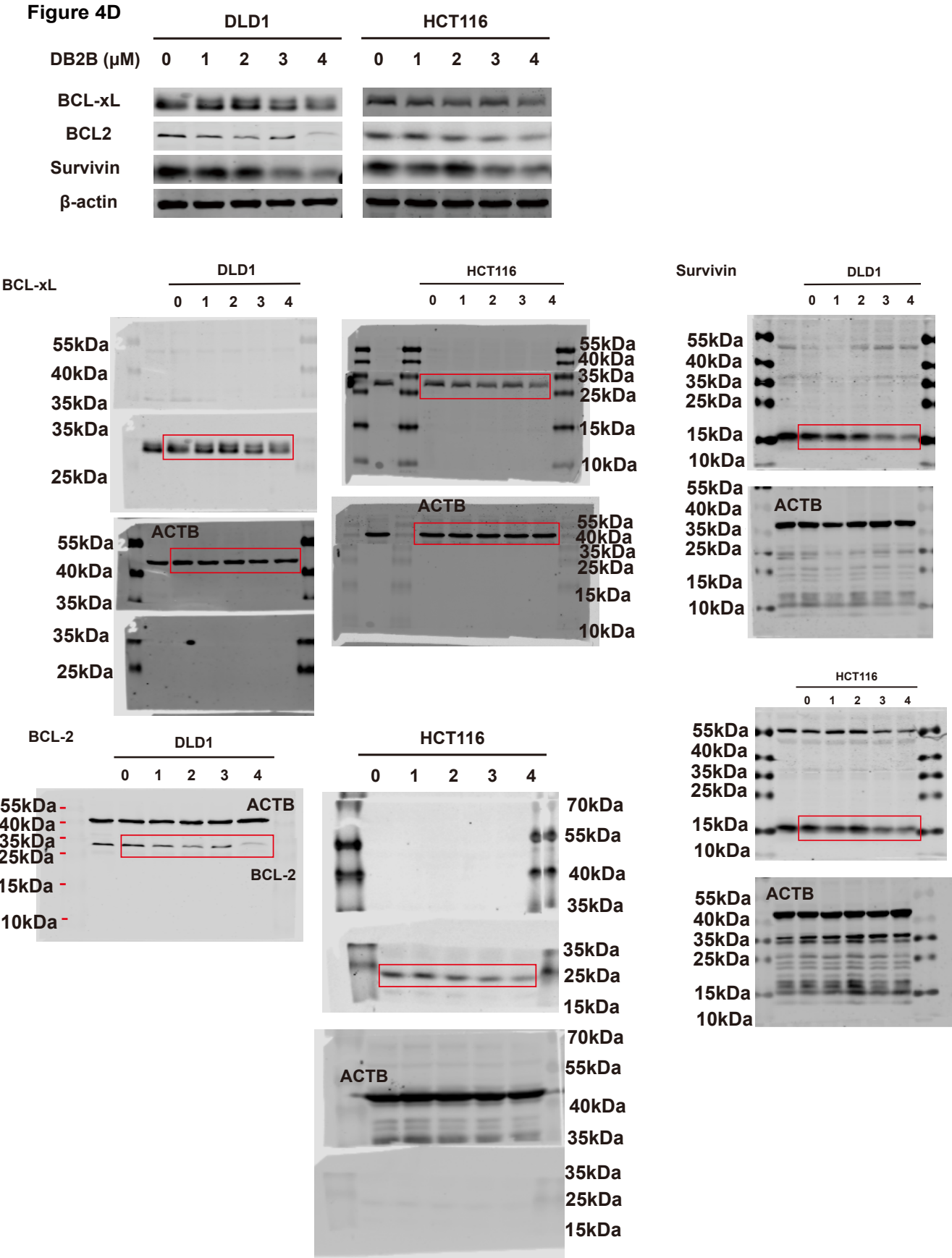

Figure 4E

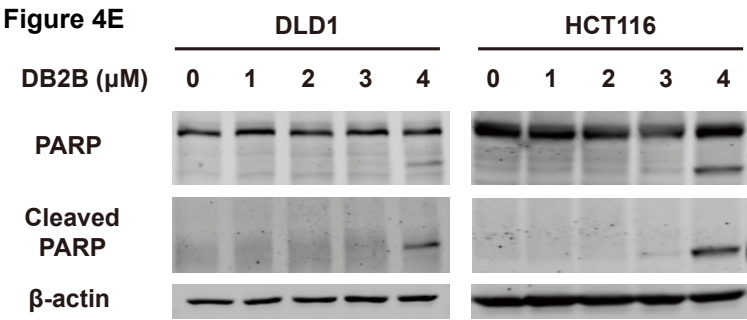

Cleaved PARP

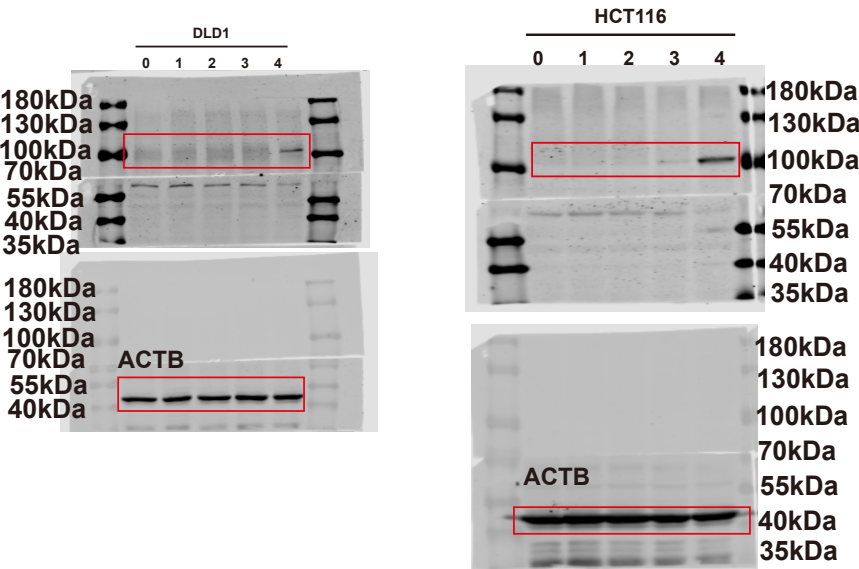

PARP

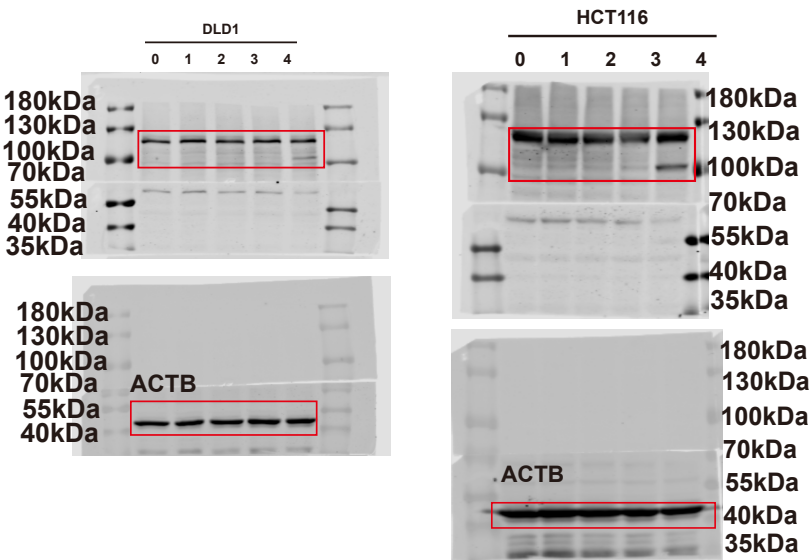

Figure 5B

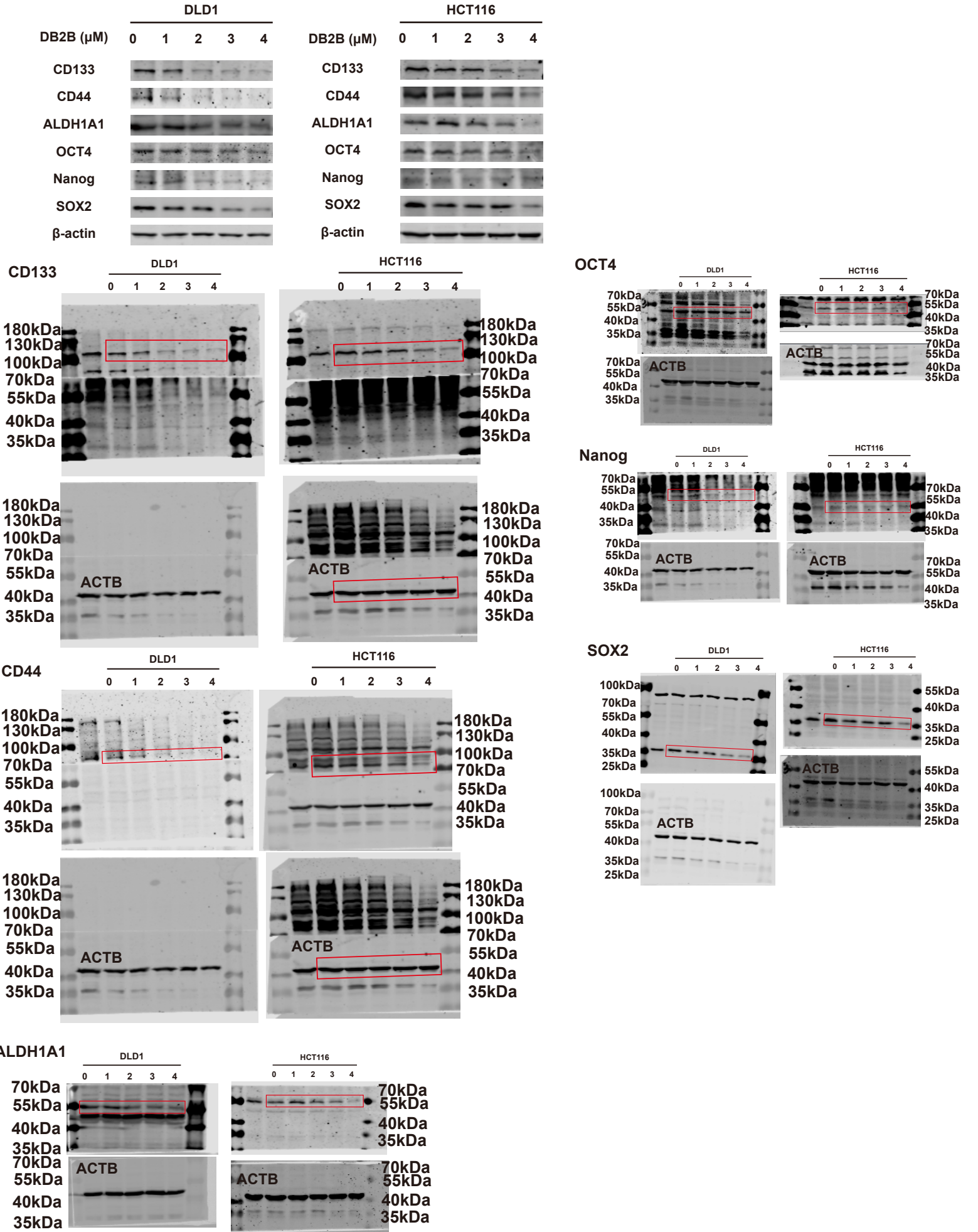

Supplement: Supplementary file 1 [file biomolecules-16-00752-s001.zip › WB original images.pdf]
